# Integration of large and diverse angiosperm DNA fragments into Asian *Gnetum* mitogenomes

**DOI:** 10.1186/s12915-024-01924-y

**Published:** 2024-06-25

**Authors:** Chung-Shien Wu, Rui-Jiang Wang, Shu-Miaw Chaw

**Affiliations:** 1 Biodiversity Research Center, Academia Sinica, Taipei, Taiwan; 2 South China Botanical Garden, Chinese Academy of Science, Guangzhou, China

**Keywords:** Gymnosperm, Angiosperm, *Gnetum*, Mitogenome, Horizontal gene transfer

## Abstract

**Background:**

Horizontal gene transfer (HGT) events have rarely been reported in gymnosperms. *Gnetum* is a gymnosperm genus comprising 25‒35 species sympatric with angiosperms in West African, South American, and Southeast Asian rainforests. Only a single acquisition of an angiosperm mitochondrial intron has been documented to date in Asian *Gnetum* mitogenomes. We wanted to develop a more comprehensive understanding of frequency and fragment length distribution of such events as well as their evolutionary history in this genus.

**Results:**

We sequenced and assembled mitogenomes from five Asian *Gnetum* species. These genomes vary remarkably in size and foreign DNA content. We identified 15 mitochondrion-derived and five plastid-derived (MTPT) foreign genes. Our phylogenetic analyses strongly indicate that these foreign genes were transferred from diverse eudicots—mostly from the Rubiaceae genus *Coptosapelta* and ten genera of Malpighiales. This indicates that Asian *Gnetum* has experienced multiple independent HGT events. Patterns of sequence evolution strongly suggest DNA-mediated transfer between mitochondria as the primary mechanism giving rise to these HGT events. Most Asian *Gnetum* species are lianas and often entwined with sympatric angiosperms. We therefore propose that close apposition of *Gnetum* and angiosperm stems presents opportunities for interspecific cell-to-cell contact through friction and wounding, leading to HGT.

**Conclusions:**

Our study reveals that multiple HGT events have resulted in massive amounts of angiosperm mitochondrial DNA integrated into Asian *Gnetum* mitogenomes. *Gnetum* and its neighboring angiosperms are often entwined with each other, possibly accounting for frequent HGT between these two phylogenetically remote lineages.

**Supplementary Information:**

The online version contains supplementary material available at 10.1186/s12915-024-01924-y.

## Background

Horizontal gene transfer (HGT) is a process that transmits genetic material between species or individuals without mating. Plant mitochondria are especially susceptible to HGT as numerous foreign mitochondrial genes have been uncovered in various seed plants [1–12]. In contrast, only a few cases of integrated mitochondrial DNA were reported in plastids [13–18]. There are three explanations for this striking disparity. First, plant mitochondria have an active transmembrane potential-dependent system that allows uptake of exogenous DNA fragments up to a few kilobase pairs [19]. Second, fusion, fission, and recombination frequently occur between plant mitochondria [20]. Third, plant mitogenomes contain lengthy intergenic regions that provide spaces for foreign DNA integration without disruption of functional genes [21].

Horizontally acquired DNA could exert a profound influence on plant mitogenome evolution. One of the most extreme examples was found in *Amborella trichopoda* whose enormous, 3.9-Mb, mitogenome contains 197 foreign mitochondrial protein genes acquired from green algae, mosses, and other angiosperms [5]. Large-scale HGT was also discovered in the *Lophophytum* mitogenome where foreign DNA accounts for more than half of the genome [22]. Acquisition of foreign genes can result in pseudogenization, replacement of native genes, or formation of chimeric genes through recombination [21]. Despite a few exceptions reported in parasitic mitogenomes [6, 23], few foreign sequences discovered in plant mitogenomes are actively expressed but rather become pseudogenes [4, 5, 7, 24]. As a result, most detectable HGT events have occurred recently, since ancient non-functional foreign genes degrade over time and vanish from contemporary mitogenomes.

HGT is thought to occur through direct physical contact and vector-mediated transmission (see review in [25]). Based on regular mitochondrial fusion in green plants, the “fusion compatibility” model was put forward to interpret transfer of massive foreign mitochondrial DNA molecules [5]. This model holds that capture of entire foreign mitochondria represents the first step toward mitochondrion-to-mitochondrion HGT, followed by mitochondrial fusion and genomic recombination to generate chimeric mitogenomes where all foreign DNA, including mitochondrial plastid-derived (MTPT) loci, are eventually acquired [5, 11, 22]. So far, horizontal acquisition of massive amounts of DNA has not been observed in any available gymnosperm mitogenome. Only three studies documented HGT of single mitochondrial loci from angiosperms to gymnosperms [2, 8, 10].

*Gnetum*, including 25‒35 species [26], is the sole genus in the family Gnetaceae of the order Gnetales (i.e., the gnetophytes). This angiosperm-like gymnosperm genus comprises mostly dioecious lianas bearing broad-bladed and pinnate-veined leaves [27–29]. They inhabit tropical and subtropical lowland rainforests of West Africa, South America, and Southeast Asia. The latter is the diversity hotspot [30]. Molecular phylogenetic studies have separated Asian *Gnetum* into two clades, I and II [2, 31], although it is still not clear whether the clade I members are monophyletic [30]. Won and Renner [2] discovered that some *Gnetum* species within the Asian clade II possess a mitochondrial *nad1* intron 2 copy with sequences nearly identical to its angiosperm homologs. This “angiosperm-type” intron was further interpreted to come from an HGT from an euasterid to *Gnetum*. Although this study represented the first discovery of HGT in gymnosperms, the following questions remain: (1) Have other HGT events taken place in *Gnetum*? (2) If yes, to what extent is the foreign DNA present in the contemporary mitogenomes? (3) Which species/genera are the HGT donors? and (4) What is the mechanism underlying *Gnetum* HGT events?

To gain a comprehensive understanding of HGT in *Gnetum*, we sequenced and assembled mitogenomes from five species that represent the two Asian clades. Our data suggest that the mitogenomes of these species have experienced multiple rounds of HGT involving large amounts of organellar DNA from diverse angiosperms. We also present evidence of DNA-mediated origins of the transferred fragments.

## Results

### Characterization of three Asian *Gnetum* mitogenomes

**Fig. 1:**
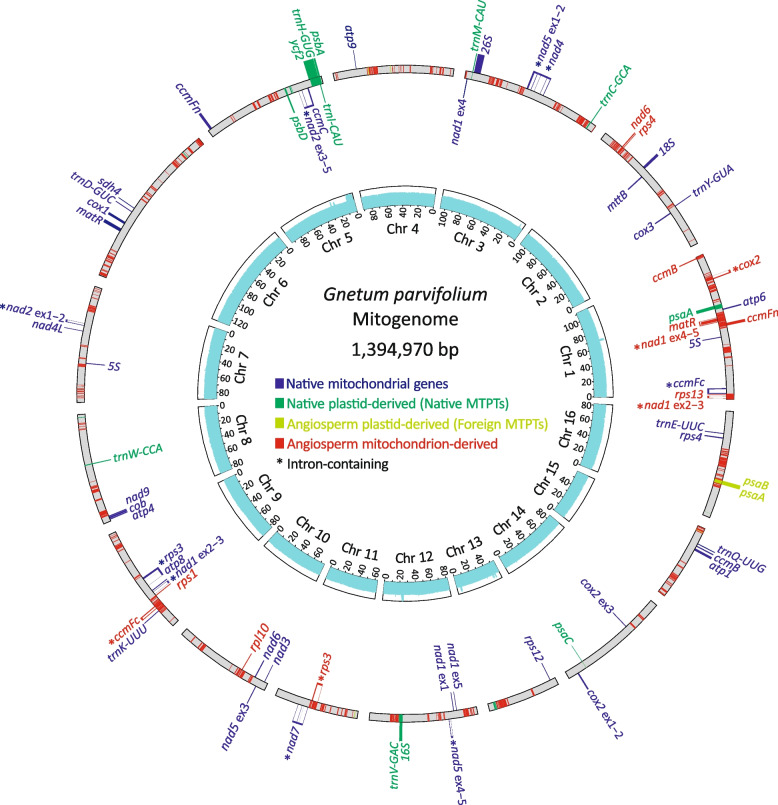
The mitogenome map of *Gnetum parvifolium*. Gray bars represent 16 circular-mapping chromosomes displayed as linear molecules for easy comparison. Genes on the outside of the chromosomes are transcribed in counterclockwise directions, while those on the inside are clockwise. Loci are color-coded depending on their origins. Light-blue histograms denote DNA read depths in log scale. Chr,  chromosome

The *G. gnemon* and *G. parvifolium* mitogenomes were assembled using the Unicycler assembler (see “Methods”) into 9 and 16 circular-mapping chromosomes with a total size of 575,501 and 1,394,970 bp, respectively (Fig. 1; Additional File 1: Fig. S1). We used a different assembler (hybridSPAdes [32]) for the *G. ula* genome because Unicycler was unable to complete this genome without system errors. We obtained 21 linear mitogenomic scaffolds for *G. ula* with a total length 1,372,030 bp (Additional File 1: Fig. S2). The GC content in these three newly assembled mitogenomes ranges from 47.2 to 48.0% (Table 1). Further, 28‒29 protein genes, 3‒4 rRNAs, and 5‒6 tRNAs are annotated as native. The native *rpl10* gene is retained in *G. gnemon* but lost from *G. parvifolium* and pseudogenized in *G. ula*. Repetitive sequences, including dispersed and tandem repeats, make up 5.5‒8.6% of the mitogenomes (Table 1). In addition, four gene clusters (i.e., *nad2* exons 3‒5 and *ccmC*; *nad2* exons 1‒2 and *nad4L*; *atp4*, *cob*, and *nad9*; *nad4* and *nad5* exons 1‒2) are conserved among the *Gnetum* mitogenomes (Fig. 1; Additional File 1: Figs. S1‒2). We detected 338‒344 C-to-U RNA editing sites, most of them (78.2‒81.6%) nonsynonymous (Table 1).

**Table 1:** Characterization of the three sequenced *Gnetum* mitogenomes

Features	*G. gnemon*	*G. parvifolium*	*G. ula*
Size (bp)	575,501	1,394,970	1,372,030
#Chromosomes/scaffolds	9	16	21
GC content (%)	48.0	47.2	47.4
Native genes^a^			
#Protein-coding genes	29	28	28
#rRNAs	4	4	3
#tRNAs	5	5	6
Native MTPTs (bp)	1693	17,002	23,210
Foreign sequences (bp)			
Angiosperm plastid-derived (foreign MTPTs)	1664	3143	6227
Angiosperm mitochondrion-derived	34,971	184,397	188,386
Repetitive sequences (bp)			
Dispersed repeats	13,132	27,632	73,036
Tandem repeats	27,212	48,950	45,332
C-to-U RNA editing			
#Nonsynonymous sites	278	269	267
#Synonymous sites	63	75	71

^a^Pseudogenes are not included.

 Like other seed plants, *Gnetum* also has mitochondrial plastid-derived DNA obtained through intracellular transfer, called native MTPTs [33]. These native MTPTs amount to 1,693‒23,210 bp (Table 1) and contain 2‒9 tRNAs that can form a three-leafed clover structure (Additional file 2: Table S1). Using blast searches, we detected numerous foreign sequences homologous to plastid or mitochondrial DNA from diverse angiosperms but not matching any non-*Gnetum* gymnosperms. According to our blast results, these foreign sequences were further designated as angiosperm plastid-derived (we call them foreign MTPTs) and angiosperm mitochondrion-derived (Table 1). Three lines of evidence indicate that these sequences are not the result of DNA contamination. First, our assembled mitogenomes contain the previously characterized “angiosperm-type *nad1* intron 2” copy that was horizontally acquired from an euasterid [2]. Second, our blast searches reveal that independently sequenced mitogenomes from other *Gnetum* species also contain homologs of most of these foreign sequences. Third, all native and foreign sequences are similar in their DNA read depths, except for the native MTPTs (Fig. 1; Additional File 1: Figs S1‒2). The latter are nearly identical in sequence to their plastid counterparts. Their reads are thus counted together with the plastid copies during read mapping, resulting in artificially higher read depths for native MTPTs than other mitogenomic regions. Collectively, the total amount of the foreign sequence varies from 36,635 to 194,613 bp, accounting for 6.4‒14.2% of the *Gnetum* mitogenomes (Table 1). This suggests that accumulation of foreign DNA has contributed to size variation among the Asian *Gnetum* mitogenomes.

### Phylogenetic evidence of HGT from eudicots to Asian *Gnetum*

**Fig. 2:**
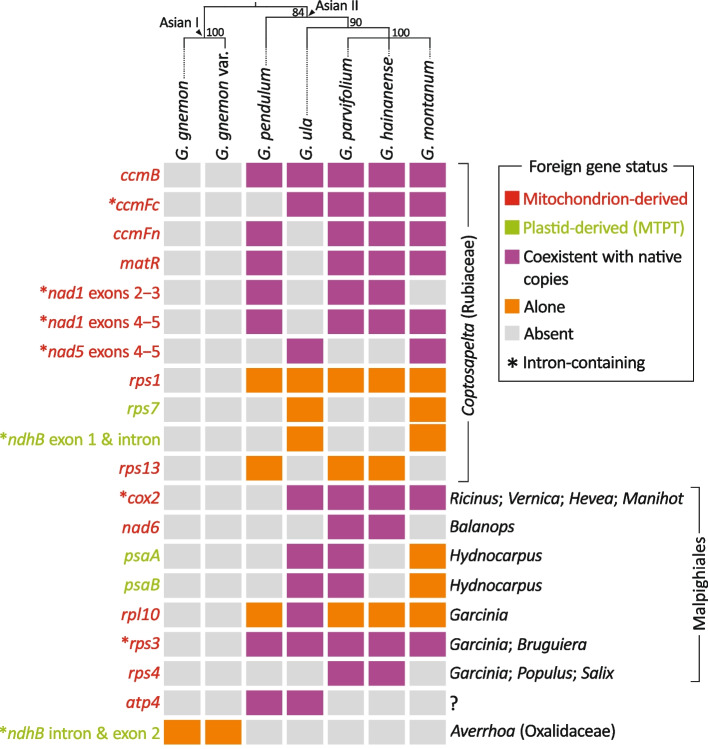
Summary of the 20 detected foreign genes and their close phylogenetic relatives. Foreign genes are listed on the left-hand side, while their close relatives are shown on the right-hand side. The color-coded rectangles in the middle denote the status of these foreign genes defined in the box of the right panel. *G. gnemon* var. *brunonianum* is abbreviated to “*G. gnemon* var.”. The ML tree shown on the upper panel was inferred from concatenating 28 native mitochondrial genes under a 50% majority rule. Supported values along branches were estimated from 1000 bootstrap replicates

We identified 20 foreign genes in the *Gnetum* mitogenomes sequenced in this study (Fig. 2). These foreign genes occupy 2.8‒11.3% of the total foreign sequences. Five of them are foreign MTPTs and the rest are derived from mitochondria. We should be able to trace the origins of these foreign genes because their homologs exist in diverse plants. We employed three strategies to broaden taxon sampling and facilitate computing. First, we sequenced and assembled two more mitogenomes from *G. gnemon* var. *brunonianum* and *G. pendulum* using short read platforms. Second, we found and annotated foreign genes from the publicly available *G. hainanense* (LC650069‒LC650085) and *G. montanum* (MW354253‒MW354270) mitogenome scaffolds. Third, all available native homologs of the 20 examined foreign genes were retrieved from ferns, gymnosperms, and angiosperms with one representative per genus.

Our maximum likelihood (ML) trees (Additional File 1: Figs. S3‒21) placed all examined foreign genes within eudicots rather than gymnosperms, strengthening the inference of horizontal acquisition of multiple genes during the Asian *Gnetum* evolution. For example, native and foreign *ccmB* genes coexist across the Asian *Gnetum* clade II (Fig. 2) and our tree suggests that all foreign *ccmB* copies form a clade that not only deviates from gymnosperms but also strongly affiliates to a Rubiaceae genus *Coptosapelta* (BS = 100%; Additional File 1: Fig. S3). Such strong affiliations were also recovered in ten other foreign genes restricted to the clade II of Asian *Gnetum*, including foreign *ccmFc*, *ccmFn*, *matR*, *nad1* exons 2‒3, *nad1* exons 4‒5, *nad5* exons 4‒5, *rps1*, *rps7*, *ndhB* exon 1 and intron, and *rps13* (all BS > 90%; Additional File 1: Figs. S4‒13). These results indicate that *Coptosapelta* is a source of ample genetic materials for *Gnetum* prior to species diversification within the Asian clade II.

Furthermore, our ML trees support multiple rather than a single origin for seven other foreign genes: *cox2*, *nad6*, *psaA*, *psaB*, *rpl10*, *rps3*, and *rps4*, though they also are confined to the Asian *Gnetum* clade II (Fig. 2). A distinctive clade was recovered for each of these seven foreign genes (Additional File 1: Figs. S14‒20), clearly indicating that their associated HGT events occurred prior to the origin of the Asian clade II. Although foreign *nad6* and *rps4* are next to each other (Fig. 1), they differ in their donors: *Balanops* for the former (BS = 98%; Additional File 1: Fig. S15), but a clade comprising *Garcinia*, *Populus*, and *Salix* for the latter (BS = 99%; Additional File 1: Fig. S20). In contrast, two neighboring foreign MTPT genes, *psaA* and *psaB*, are of a congeneric origin from *Hydnocarpus* (Both BS > 95%; Additional File 1: Figs. S16‒17), indicating a co-transfer event. Foreign *cox2*, *rpl10*, and *rps3* genes reside on different chromosomes and have independent origins (Fig. 2): the clade comprising *Ricinus*, *Vernica*, *Hevea*, and *Manihot* for foreign *cox2* (BS = 86%; Additional File 1: Fig. S14), only *Garcinia* for foreign *rpl10* (BS = 91%; Additional File 1: Fig. S18), and the *Garcinia*-*Bruguiera* clade for foreign *rps3* (BS = 83%; Additional File 1: Fig. S19). Notably, the abovementioned ten genera belong to Malpighiales (Fig. 2), highlighting frequent HGT from this eudicot order to Asian *Gnetum*.

The foreign *atp4* copies in *G. ula* and *G. pendulum* were not placed into any sister clade with greater than 50% confidence (Fig. 2; Additional File 1: Fig. S21). A foreign MTPT that includes *ndhB* intron and its downstream exon (i.e., *ndhB* intron and exon 2) is uniquely present in *G. gnemon* and its variety within the Asian clade I (Fig. 2). This *G. gnemon*-specific locus has a close relationship to *Averrhoa* of Oxalidaceae (BS = 73%; Additional File 1: Fig. S12) but differs from its *Coptosapelta*-derived homolog in *G. ula* and *G. montanum* within the Asian clade II (Fig. 2). Therefore, there were at least two independent transfers of *ndhB* from different eudicots to Asian *Gnetum* in the past. Taken together, our phylogenetic results provide solid evidence that multiple rounds of independent HGT events have significantly shaped the mitogenome complexity in Asian *Gnetum*.

### Foreign genes in Asian *Gnetum* mitogenomes are non-functional

Among the 15 foreign mitochondrion-derived genes (Fig. 2), 12 coexist with functional native homologs and the rest are either alone or present with a pseudogenized native copy (e.g.*, rpl10* of *G. ula*). This prompts us to raise the question: Are foreign genes transcribable to functionally complement the lost native homologs?

Combining the mapped RNA reads from both strands covers more than 91% of the three mitogenomes we sequenced. Focusing on *G. parvifolium* and *G. ula* because their mitogenomes contain many foreign genes, we calculated transcripts per million (TPM) values for all genes in their mitogenomes. TPM values of the foreign genes range from 2.7 to 614.3 in *G. parvifolium* and 36.3 to 561.9 in *G. ula* (Additional File 1: Fig. S22). No C-to-U edited sites were found in the RNA reads mapped to these foreign genes. In contrast, native genes exhibit much higher TPM values, ranging between 3438.6 and 97,600.5 in *G. parvifolium* and 2619.8 to 142,232.2 in *G. ula*, a statistically significant difference (two-tailed Mann–Whitney *U* test, *P* < 0.01 for both *G. parvifolium* and *G. ula*). Given that >91% of the *Gnetum* mitogenomes are transcribable, some level of transcription is expected for non-functional loci. Coupled with the presence of premature termination codons and frame-shifting indels in these genes, we consider it likely that the foreign copies are not functional.

### DNA-mediated HGT in Asian *Gnetum* mitochondria

It is worth noting that 8 of the 20 foreign genes contain introns, including *ccmFc*, *nad1* exons 2‒3, *nad1* exons 4‒5, *nad5* exons 4‒5, *ndhB* exon 1 and intron, *cox2*, *rps3*, and *ndhB* intron and exon 2 (Figs. 1‒2; Additional File 1: Figs. S1‒2). The foreign *nad1* exons 4‒5 gene contains foreign *matR* within its intron (Fig. 1). Their congeneric origin is phylogenetically supported (Fig. 2). Apparently, these eight foreign intron-containing genes and *matR* have originated from DNA-mediated transfer. The clustering of foreign genes is a hallmark of DNA-mediated transfer when all foreign genes within a cluster have the same phylogenetic origin [7]. Such traits are observed in three foreign gene clusters in *G. parvifolium* (i.e., *nad1* exons 2‒3 and *rps13*; *ccmFn* and *nad1* exons 4‒5; *rps1* and *ccmFc*) and two in *G. ula* (i.e., *psaA* and *psaB*; *nad5* exons 4‒5, *rps7*, and *ndhB* exon 1 and intron).

**Fig. 3:**
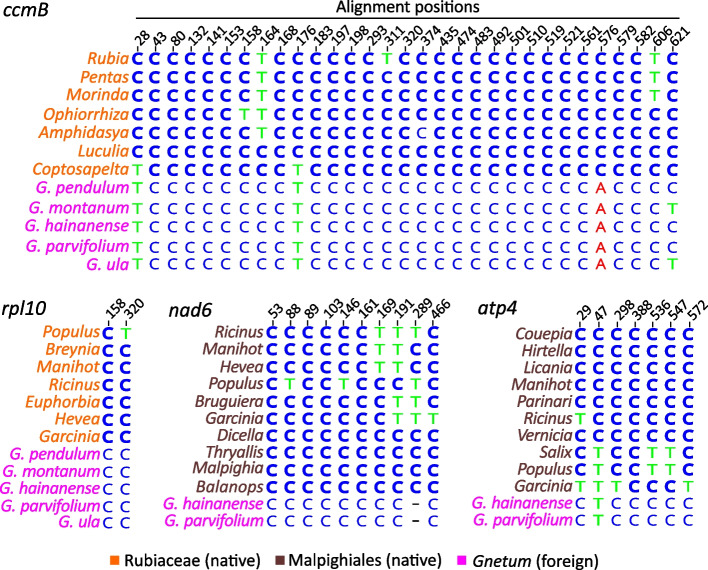
Nucleotide states of RNA editing sites in the foreign genes. Cytidines in native genes are bolded if their transcripts are predicted to be C-to-U edited. Only conserved editing sites on the gene alignment are shown

The prevalence of C-to-U RNA editing in seed plant mitochondria provides traceable information to distinguish retroprocessing from direct integration of genomic DNA [4]. Four foreign genes, *ccmB*, *rpl10, nad6*, and *rps4*, were examined because the former two lack introns and the latter two have different origins despite forming a gene cluster (Figs. 1 and 2). If integration was mediated by mRNA, we would expect to see thymidines at the conserved editing sites of foreign genes. However, we observe mostly unedited cytidines at these loci (Fig. 3), making the cDNA integration origin unlikely. Moreover, we find thymidines at positions 28 and 176 of the *ccmB* alignment in both *Gnetum* and *Coptosapelta* but cytidines in other Rubiaceae genera (Fig. 3). This finding reinforces the transfer of *ccmB* from *Coptosapelta* to *Gnetum* after losses of these two editing sites through genomic C-to-T substitutions in *Coptosapelta*.

### Ancient HGT residues in Asian *Gnetum* mitogenomes

**Fig. 4:**
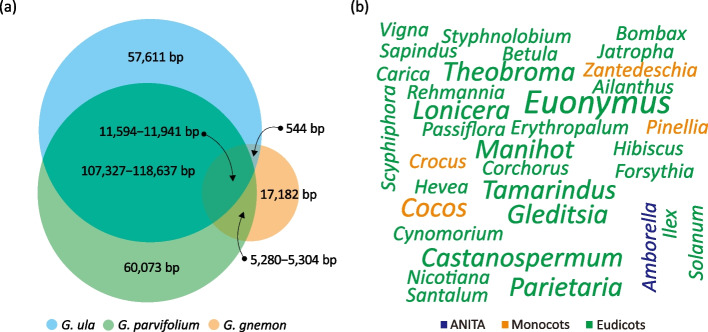
Ancient angiosperm mitochondrion-derived sequences. **a** Quantitative Venn diagrams indicate the number of unique and shared angiosperm mitochondrion-derived sequences among *G. gnemon*, *G. parvifolium*, and *G. ula*. **b** Word clouds show the best matched taxa with the font size reflecting their frequencies in the blast searches of the 52 ancient angiosperm mitochondrion-derived sequences (also see Additional file 2: Table S2)

The 20 foreign genes mentioned above are relatively young since they are either Asian clade I- or II-specific (Fig. 2). To investigate ancient HGT residues, we looked for angiosperm mitochondrion-derived sequences shared by the two Asian *Gnetum* clades. We identified 544 bases shared by *G. gnemon* and *G. ula*, 5280‒5304 by *G. gnemon* and *G. parvifolium*, and 11,594‒11,941 by all three species (Fig. 4a). These shared bases constitute 45 separate non-coding sequences in *G. gnemon*, varying from 61 to 1526 bp in length (Additional file 2: Table S2). Blast searches of these 45 sequences reveal that (1) they are not homologous to any available mitogenomic sequences from non-*Gnetum* gymnosperms and that (2) except for *Gnetum* itself, the best matches are from mitogenomes of diverse angiosperms, such as the ANITA group, monocots, and eudicots (Fig. 4b). Despite the absence of phylogenetic evidence, these 45 foreign sequences can be regarded as ancient HGT residues gained before the split of the two Asian clades.

## Discussion

Previously, exploration of foreign genes in gymnosperm mitogenomes has mainly relied on PCR [2, 8, 10]. Despite easy management, PCR methods have limitations in evaluating foreign genes. First, it is difficult to design appropriate primers when the donors of targeted foreign genes are uncertain. Second, most foreign genes are pseudogenized and degraded over time, impeding the design of universal primers across a taxonomic range of interest. Third, the exact cellular compartments where foreign genes of PCR targets reside need verification. Thus, PCR-based methods can underestimate the foreign (especially non-coding) DNA content in a genome.

In this study, we sequenced and assembled the mitogenomes from five *Gnetum* species across two Asian clades. Our genome-scale survey reveals that Asian *Gnetum* has received tremendous amount of foreign DNA, leading to great variation in their mitogenome size. Despite highly variable, the numbers of foreign genes found in the Asian *Gnetum* clade II are unprecedented among the so far elucidated gymnosperm mitogenomes. This observation provides evidence that gymnosperm mitogenomes can carry a large number of exogenous DNA/genes, like some angiosperms, such as *Amborella* [5], *Geranium* [7], and several parasitic eudicots [6, 9, 11, 22].

All 20 foreign genes in the examined *Gnetum* mitogenomes are pseudogenized and expressed at significantly lower levels than native loci without RNA editing. Together with the presence of foreign introns and the clustering of foreign genes of the same origin, these observations indicate DNA-mediated transfer from diverse angiosperms to Asian *Gnetum* as a consequence of silent HGT [21]. We see no evidence of RNA-mediated HGT during the *Gnetum* mitogenome evolution. This agrees well with several earlier studies showing that transfer of foreign DNA fragments is overwhelmingly common among seed plant mitochondria [4, 5, 7, 11, 22]. Furthermore, we detected many ancient angiosperm mitochondrion-derived sequences shared by both Asia *Gnetum* clades but not by any non-*Gnetum* gymnosperms. Two alternative hypotheses can explain their origins: (1) they were originally ancient angiosperm mitochondrial DNA fragments independently inserted into the common ancestor’s mitogenomes of the two Asia *Gnetum* clades or (2) they were initially seed plant descendants but had been lost from all gymnosperms except *Gnetum*. The second hypothesis is unlikely because *Gnetum* mitogenomes have drastically elevated rates of nucleotide substitutions with frequent gene loss [34, 35].

Eleven of the 20 foreign genes we identified were phylogenetically inferred to be horizontally transferred from *Coptosapelta* to the ancestors of the Asian *Gnetum* clade II (Fig. 2). This inference is supported by the emergence of *Coptosapelta* during the Upper Cretaceous [36], much earlier than the split of the two Asian *Gnetum* clades dating around 10‒39 MYA [31]. *Coptosapelta* is found in the southeast of Asia where species of the Asian *Gnetum* clade II also grow. Such an overlapping biogeographic distribution creates opportunities for HGT between these phylogenetically remote seed plant lineages. Hence, the most parsimonious HGT paradigm is that a large piece of *Coptosapelta* mitochondrial DNA invaded the ancestral mitogenomes of the Asian *Gnetum* clade II. The subsequent mitogenomic rearrangement and species-specific degradation explain the patchy distribution of the *Coptosapelta*-derived genes in the Asian *Gnetum* clade II ([2]; this study). This co-transfer paradigm also holds for the acquisition of the *Coptosapelta*-derived *rps7* and *ndhB* exon 1 and intron cluster via mitochondrion-to-mitochondrion HGT after intracellular plastid-to-mitochondrion transfer of the associated genes in *Coptosapelta*. Indeed, a strong affinity of *Gnetum* to Rubiaceae (to which *Coptosapelta* belongs) is revealed in the phylogenetic tree based on the flanking regions of this *Coptosapelta*-derived MTPT gene cluster and its mitochondrial homologs (Additional File 1: Fig. S23).

In contrast, several Malpighiales genera appear to be donors of seven other foreign genes also specific to the Asian *Gnetum* clade II. However, the Malpighiales ancestry of these foreign genes cannot be supported because the crown groups of Malpighiales diverged approximately 90 MYA [37]. It is also possible that the origin of these foreign genes was mis-inferred in our trees due to a biased taxon sampling resulting from under-representation of some angiosperm lineages in GenBank. Nonetheless, the discrete chromosomal locations coupled with distinct phylogenetic origins lead us to believe that these Malpighiales-derived genes initially came from independent HGT events, especially since they also include a foreign MTPT gene cluster *psaA*-*psaB*. Although the flanking region sequences proved uninformative (Additional File 1: Fig. S24), we found homologs of this *psaA*-*psaB* cluster in the mitogenomes of some Malpighiales species, such as *Populus tremula* (NC028096), *Hevea pauciflora* (NC080334), and *Kandelia obovata* (NC06922). Therefore, it is reasonable to conclude that integration of the Malpighiales-derived *psaA*-*psaB* cluster in Asian *Gnetum* has resulted from mitochondrion-to-mitochondrion HGT, resembling the majority of foreign MTPTs in angiosperms [33]. Unfortunately, we could not clarify the transfer route for the *G. gnemon*-specific foreign MTPT (i.e., *ndhB* intron and exon 2) because homologous sequences of this MTPT’s flanking region are not present in GenBank.

Why dose Asian *Gnetum* stand out for the unusually large amounts of foreign genes in their mitogenomes? Strikingly, some conifer mitogenomes are huge with lengthy intergenic regions, offering more spaces for foreign gene integration. The *Larix sibirica* mitogenome is 8‒20-fold larger than *Gnetum*, yet no foreign genes have been reported in this conifer species [38]. Insect-mediated HGT during pollination was previously suggested as a possible mechanism for uptake of angiosperm DNA in *Gnetum* mitogenomes [2]. However, illegitimate pollination is more likely when two plant species are closely related [25].

**Fig. 5:**
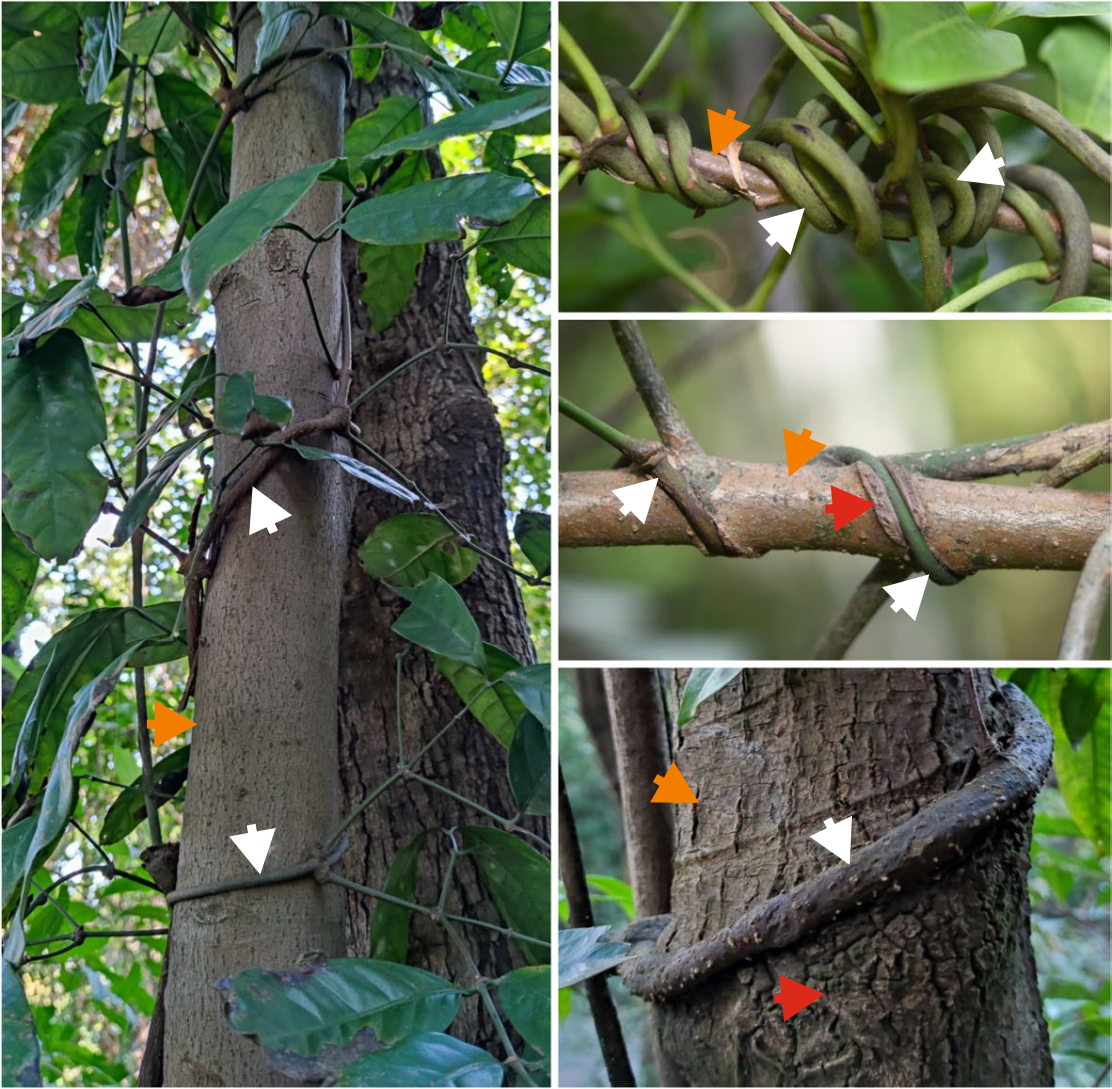
Photos showing closely twisted *Gnetum* and angiosperms. White, orange, and red arrows indicate *Gnetum*, angiosperms, and healed wounds, respectively

Rice et al. [5] proposed a “wounding-HGT model” and maintained that wounds facilitate capture of foreign mitochondria whose genomic DNA is then integrated into the recipient mitogenome via mitochondrial fusion. Cell-to-cell movement of mitochondria and the subsequent fusion also can be responsible for horizontal mitochondrial DNA transfer between grafted plants [39]. Formation of channels that allow intercellular exchange of organelles was observed in direct contact between callus cells generated from plant grafted junctions [40]. *Gnetum* is the only lianas among gymnosperms. Their stems and those of sympatric angiosperms are often closely entwined (Fig. 5). Due to frequent friction from the entwined stems between *Gnetum* and angiosperms, wounds can be easily formed, creating opportunities for interspecific cell-to-cell contact and exchange of mitochondria. The large-scale integration of angiosperm mitochondrial DNA in the Asian *Gnetum* clade II mitogenomes, including foreign MTPTs, can be explained by mitochondrial fusion after capture of foreign mitochondria. Vertical transfer of the integrated foreign DNA could be achieved if the transgenic cell is incorporated into meristems that later differentiate into reproductive tissues [25, 41]. Future work will have to clarify whether the ancestry of the Asian *Gnetum* clade II had encountered strong genetic bottlenecks to fix the integrated foreign DNA in its populations.

## Conclusions

Two decades after the discovery of the angiosperm-type *nad1* intron 2 [2] in *Gnetum*, we provide the first evidence that horizontal transfer of massive amounts of angiosperm mitochondrial DNA has had a great influence on Asian *Gnetum* mitogenome evolution. The high prevalence of foreign DNA makes Asian *Gnetum* an excellent system for investigating HGT between gymnosperms and other seed plants. The detection of multiple HGT events suggests that there was an active mechanism allowing for the frequent transfer of mitochondrial DNA from diverse angiosperms to the Asian *Gnetum* ancestry. The DNA-mediated HGT of multiple mitochondrial genes suggests that interspecific mitochondrial fusions may account for the high prevalence of angiosperm mitochondrion-derived DNA in the Asian *Gnetum* mitogenomes, given that *Gnetum* and angiosperms are often closely entwined to facilitate direct cell-to-cell contact between species. Deciphering the mitogenomes from African and South American *Gnetum* and other species from the Asian *Gnetum* clade I will help reconstruct a more comprehensive HGT history in *Gnetum*.

## Methods

### Taxon sampling, DNA and RNA extraction, and sequencing

Fresh leaves were harvested from *G. gnemon*, *G. parvifolium*, and *G. ula* individuals grown in the Academia Sinica greenhouse. Genomic DNAs were extracted from harvested leaves using the CTAB method described in [42]. Short DNA fragments were removed using Short Fragment Eliminator Kits (Circulomics, MD), followed by library preparation for MinION sequencers (FLO-MIN106, Oxford Nanopore Technologies: ONT) based on the recommend protocol with native barcoding genomic DNAs (EXP-NBD104), 1D sequencing kit, and SQK-LSK109 ligation kit. Approximately 13‒15 million long reads were generated for each species. Over 4 million pairs of 150 bp pair-end (PE) reads per species were also obtained from an Illumina NovaSeq 6000 platform and DNA libraries constructed using Celero™ DNA-Seq Library Preparation Kits (TECAN, Switzerland). Genomic DNA of *G. gnemon* var. *brunonianum* and *G. pendulum* collected in the South China Botanical Garden of the Chinese Academy of Sciences was extracted using DNeasy Plant Pro and Plant Kits (QIAGEN, Germany) and sequenced on a HiSeq X Ten system to yield over 8 million pairs of 100 bp PE reads per species. The sampled specimens are deposited in the herbarium with voucher numbers shown within parentheses: *G. gnemon* (Chaw1605), *G. parvifolium* (Chaw1607), and *G. ula* (Chaw1608) in Academia Sinica; *G. pendulum* (YN2024001) and *G. gnemon* var. *brunonianum* (YN2024002) in the South China Botanical Garden of Chinese Academy of Science. Total RNAs were isolated from fresh young *G. gnemon*, *G. parvifolium*, and *G. ula* leaves using Plant Total RNA Purification Kits (GeneMark, Georgia). After DNase I treatment, the extracted RNA was subjected to rRNA depletion and strand-specific library construction using Ovation RNA-Seq Systems 1‒16 for Model Organisms‒*Arabidopsis* (NuGEN, CA). The RNA libraries were sequenced on a NovaSeq 6000 platform to produce approximately 4.1‒5.5 million pairs of 150 bp PE reads per species.

### Mitogenome assembly and annotation

Before de novo genome assembly, ONT reads shorter than 5 kb were discarded to decrease the computational burden. We used the Unicycler v0.5.0 hybrid assembler [43] that incorporates PE and ONT reads to assemble the *G. gnemon* and *G. parvifolium* gnomes. After short read assembly with a wide range of *k*-mer sizes (min_kmer_frac = 0.6; kmer_count = 5), Unicycler evaluated each of these assembly graphs and then used long reads to build bridges. The nature of multiple genome copies per cell enables distinguishing mitochondrial scaffolds from most of the nuclear ones in genome skimming data [44]. We set the options “min_fasta_length = 2000” and “--spades_options --cov-cutoff 2” to remove potential nuclear contigs and to reduce computational complexity. Another hybrid assembler, hybridSPAdes [32], was adopted for *G. ula* because system errors repeatedly occurred when application of Unicycler to this species. PE reads from *G. gnemon* var. *brunonianum* and *G. pendulum* were assembled using SPAdes v3.13 [45] with options of “careful” and *k*-mer sizes 21, 33, 55, 77, and 89. Mitochondrial chromosomes/scaffolds were searched by blast against protein-coding genes and rRNAs retrieved from *Cycas* (NC010303) and *Ginkgo* (NC027976) mitogenomes under an expected threshold = 10^-10^. The identified mitochondrial chromosomes/scaffolds were polished using their associated PE reads in Pilon v1.24 [46] with two to three rounds of iterations. Genome annotations were conducted in Geneious Prime [47] with the *Ginkgo* mitogenome as the reference. tRNAs were predicted using tRNAscan-SE v2.0 [48].

### Exploration of MTPT and foreign sequences

To explore MTPT and foreign sequences, mitochondrial chromosomes/scaffolds were clipped into 1-kb non-overlapping fragments using the “getfasta” program implemented in Bedtools v2.31.0 [49]. These 1-kb fragments were subsequently megablasted against the NCBI nr/nt database with the parameters: max targets = 100, max E-value = 10^-10^, and word size = 28. Queries were recognized as native MTPTs if they matched published *Gnetum* plastomes well. MTPTs were considered foreign if they matched only non-gymnosperm plastomes. We considered other mitochondrial loci foreign if the sequences they matched were not included in mitogenomes of non-*Gnetum* gymnosperms. To identify shared foreign loci in the *Gnetum* mitogenomes, we first searched syntenic regions among *G. gnemon*, *G. parvifolium*, and *G. ula* using MUMmer 3.0 [50], and then compared foreign loci within syntenic regions between species.

### Read mapping, RNA editing site detection, and TPM calculation

DNA read mapping analyses were performed using Bowtie2 v2.5.1 [51] with the default settings. Read depths were counted in Geneious Prime, followed by transformation into log scales: Log (coverage + 1) / log (maximum coverage + 1). For detection of RNA editing sites and calculation of gene expression levels, strand-specific RNA reads were mapped to the assembled *Gnetum* mitogenomes using TopHat v2.1.1 [52] with the parameters: library-type = fr-secondstrand, read-mismatches = 15, read-gap-length = 0, and read-edit-dist = 15. Samtools 1.9 [53] was used to filter, sort, and combine the mapped RNA reads into BAM files. These files were then used to identify RNA editing sites using the method described in Wu and Chaw [35]. Transcripts generated from native and foreign genes were also counted in Geneious Prime and then normalized into TPM values. We used PREPACT v3.12 [54] to predict RNA editing sites in angiosperm mitochondrial genes homologous to the foreign genes identified in *Gnetum* with the options of “Type of analyses = BLASTX prediction” and “Protein reference = Organelle: mitochondrion”, such that all angiosperm references were selected.

### Identification of HGT origins for foreign genes

To trace HGT origins, foreign genes were extracted from the identified foreign sequences. These foreign genes were aligned with their native homologs, including MTPTs, using MAFFT v7.490 [55], followed by manual adjustments. Gene trees were estimated using IQ-TREE v2.2.0 [56] with 5000 ultrafast bootstraps and the “MFP” option of automatic model selection. Possible HGT donors were recognized if the examined foreign genes were placed as a sister to a particular angiosperm clade with >50% bootstrap support. Trees were visualized in Figtree v1.4.4 [57].

### Mitogenome visualization

Mitogenome maps were drawn using Circos v0.67 [58].

### Supplementary Information

Additional file 1: Figs S1‒S24. Fig. S1. The mitogenome map of *Gnetum gnemon*. Grey bars represent nine circular-mapping chromosomes that are displayed as linear molecules for easy comparisons. Loci are color-coded depending on their origins. Light-blue histograms denote DNA read depths in log scales. Chr, chromosome. Fig. S2. The draft mitogenome map of *Gnetum ula*. Grey bars represent 21 linear scaffolds. Loci are color-coded depending on their origins. Light-blue histograms denote DNA read depths in log scales. SC, scaffold. Fig. S3. A ML tree inferred from *ccmB* using ferns as the outgroup. Subtrees within left boxes indicate the relative placements of foreign and native *ccmB* genes in *Gnetum* with bootstrap values under a 50% majority rule. Fig. S4. A ML tree inferred from *ccmFc* using ferns as the outgroup. Subtrees within left boxes indicate the relative placements of foreign and native *ccmFc* genes in *Gnetum* with bootstrap values under a 50% majority rule. Fig. S5. A ML tree inferred from *ccmFn* using ferns as the outgroup. Subtrees within left boxes indicate the relative placements of foreign and native *ccmFn* genes in *Gnetum* with bootstrap values under a 50% majority rule. Fig. S6. A ML tree inferred from *matR* using ferns as the outgroup. Subtrees within left boxes indicate the relative placements of foreign and native *matR* genes in *Gnetum* with bootstrap values under a 50% majority rule. Fig. S7. A ML tree inferred from *nad1* exons 2‒3 using ferns as the outgroup. Subtrees within left boxes indicate the relative placements of foreign and native *nad1* exons 2‒3 loci in *Gnetum* with bootstrap values under a 50% majority rule. Fig. S8. A ML tree inferred from *nad1* exons 4‒5 using ferns as the outgroup. Subtrees within left boxes indicate the relative placements of foreign and native *nad1* exons 4‒5 loci in *Gnetum* with bootstrap values under a 50% majority rule. Fig. S9. A ML tree inferred from *nad5* exons 4‒5 using ferns as the outgroup. Subtrees within left boxes indicate the relative placements of foreign and native *nad5* exons 4‒5 loci in *Gnetum* with bootstrap values under a 50% majority rule. Fig. S10. A ML tree inferred from *rps1* using ferns as the outgroup. The subtree indicates the relative placement of foreign *rps1* genes in *Gnetum* with bootstrap values under a 50% majority rule. The native *rps1* has been lost from *Gnetum* and thus was not included in this tree. Fig. S11. A ML tree inferred from plastid (native) and mitochondrial plastid-derived (foreign) *rps7* using ferns as the outgroup. Note that *Gnetum*’s plastid loci are separated from their mitochondrial homologs, suggesting that the latter are HGT loci rather than MTPTs. Subtrees detail the relative placements of foreign and native *rps7* genes in *Gnetum* with bootstrap values under a 50% majority rule. Fig. S12. A ML tree inferred from plastid (native) and mitochondrial plastid-derived (foreign) *ndhB* using ferns as the outgroup. The two remote clades in *Gnetum* suggest that two independent HGT events have taken place in the Asia clades I and II, respectively. Subtrees detail the relative placements of foreign *ndhB* loci in *Gnetum* with bootstrap values under a 50% majority rule. The plastid *ndhB* has been lost from *Gnetum* and thus was not included in this tree. Fig. S13. A ML tree inferred from *rps13* using ferns as the outgroup. The subtree details the relative placements of foreign *rps13* genes in *Gnetum* with bootstrap values under a 50% majority rule. The native *rps13* has been lost from *Gnetum* and thus was not included in this tree. Fig. S14. A ML tree inferred from *cox2* using ferns as the outgroup. Subtrees show the relative placements of foreign and native *cox2* gene in *Gnetum* with bootstrap values under a 50% majority rule. Fig. S15. A ML tree inferred from *nad6* using ferns as the outgroup. Subtrees show the relative placements of foreign and native *nad6* genes in *Gnetum* with bootstrap values under a 50% majority rule. Fig. S16. A ML tree inferred from plastid, native MTPT, and foreign MTPT copies of *psaA* using ferns as the outgroup. Subtrees detail the relative placements of these three *psaA* copies in *Gnetum* with bootstrap values under a 50% majority rule. Fig. S17. A ML tree inferred from plastid, native MTPT, and foreign MTPT copies of *psaB* using ferns as the outgroup. Subtrees detail the relative placements of these three *psaB* copies in *Gnetum* with bootstrap values under a 50% majority rule. Fig. S18. A ML tree inferred from *rpl10* using ferns as the outgroup. Subtrees detail the relative placements of foreign and native *rpl10* genes in *Gnetum* with bootstrap values under a 50% majority rule. The native *rpl10* has been lost from all sampled *Gnetum* species within the Asia clade II, except for *G. ula* whose *rpl10* is retained but pseudogenized. Fig. S19. A ML tree inferred from *rps3* using ferns as the outgroup. Subtrees show the relative placements of foreign and native *rps3* genes in *Gnetum* with bootstrap values under a 50% majority rule. Fig. S20. A ML tree inferred from *rps4* using ferns as the outgroup. Subtrees detail the relative placements of foreign and native *rps4* genes in *Gnetum* with bootstrap values under a 50% majority rule. Fig. S21. A ML tree inferred from *atp4* using ferns as the outgroup. Subtrees indicate the relative placements of foreign and native *atp4* in *Gnetum* with bootstrap values under a 50% majority rule. In *Gnetum*, the close relative to foreign *atp4* is uncertain, so that the possible donor of this foreign gene is designated as questionable (“?”) in Fig. 2. Fig. S22. Comparison of mitochondrial RNA expression level between foreign and native genes in *G. parvifolium* and *G. ula*. Fig. S23. A ML tree inferred from the flanking region of the 3’ end of the *Coptosapelta*-derived *ndhB* exon1 & intron locus and its mitochondrial homologies. The tree is condensed under a 50% majority rule. Fig. S24. A ML tree inferred from the flanking region of the 3’end of the Malpighiales-derived *psaB* locus and Its mitochondrial homologies. The tree is condensed under a 50% majority rule.

Additional file 2: Tables S1‒S2. Table S1. tRNAs from native MTPTs. Table S2. Angiosperm mitochondrion-derived sequences shared by the two Asian *Gnetum* clades.

## Data Availability

Raw sequence data is available through the NCBI SRA under BioProject accessions: PRJNA1029351, PRJNA1029324, and PRJNA1029334 [59–61]. The annotated mitochondrial chromosomes/scaffolds are deposited in the GenBank under accessions: LC783565‒LC783658 [62].

## Abbreviations

HGT: Horizontal gene transfer
MTPT: Mitochondrial plastid-derived locus

## References

[1] Bergthorsson U, Adams KL, Thomason B, Palmer JD (2003). Widespread horizontal transfer of mitochondrial genes in flowering plants. Nature..

[2] Won H, Renner SS (2003). Horizontal gene transfer from flowering plants to *Gnetum*. Proc Natl Acad Sci U S A..

[3] Davis CC, Wurdack KJ (2004). Host-to-parasite gene transfer in flowering plants: phylogenetic evidence from Malpighiales. Science..

[4] Mower JP, Stefanović S, Hao W, Gummow JS, Jain K, Ahmed D (2010). Horizontal acquisition of multiple mitochondrial genes from a parasitic plant followed by gene conversion with host mitochondrial genes. BMC Biol..

[5] Rice DW, Alverson AJ, Richardson AO, Young GJ, Sanchez-Puerta MV, Munzinger J (2013). Horizontal transfer of entire genomes via mitochondrial fusion in the angiosperm *Amborella*. Science..

[6] Xi Z, Wang Y, Bradley RK, Sugumaran M, Marx CJ, Rest JS (2013). Massive mitochondrial gene transfer in a parasitic flowering plant clade. PLoS Genet..

[7] Park S, Grewe F, Zhu A, Ruhlman TA, Sabir J, Mower JP (2015). Dynamic evolution of *Geranium* mitochondrial genomes through multiple horizontal and intracellular gene transfers. New Phytol..

[8] Wang B, Climent J, Wang XR. Horizontal gene transfer from a flowering plant to the insular pine *Pinus canariensis* (Chr. Sm. Ex DC in Buch). Heredity. 2015;114(4):413‒18. 10.1038/hdy.2014.118 PMC4359980 25604946

[9] Sanchez-Puerta MV, García LE, Wohlfeiler J, Ceriotti LF (2017). Unparalleled replacement of native mitochondrial genes by foreign homologs in a holoparasitic plant. New Phytol..

[10] Forgione I, Bonavita S, Regina TMR (2019). Mitochondria of *Cedrus atlantica* and allied species: a new chapter in the horizontal gene transfer history. Plant Sci..

[11] Roulet ME, Garcia LE, Gandini CL, Sato H, Ponce G, Sanchez-Puerta MV (2020). Multichromosomal structure and foreign tracts in the *Ombrophytum subterraneum* (Balanophoraceae) mitochondrial genome. Plant Mol Biol..

[12] Choi KS, Park S (2021). Complete plastid and mitochondrial genomes of *Aeginetia indica* reveal intracellular gene transfer (IGT), horizontal gene transfer (HGT), and cytoplasmic male sterility (CMS). Int J Mol Sci..

[13] Iorizzo M, Senalik D, Szklarczyk M, Grzebelus D, Spooner D, Simon P (2012). De novo assembly of the carrot mitochondrial genome using next generation sequencing of whole genomic DNA provides first evidence of DNA transfer into an angiosperm plastid genome. BMC Plant Biol..

[14] Straub SC, Cronn RC, Edwards C, Fishbein M, Liston A (2013). Horizontal transfer of DNA from the mitochondrial to the plastid genome and its subsequent evolution in milkweeds (Apocynaceae). Genome Biol Evol..

[15] Ma PF, Zhang YX, Guo ZH, Li DZ (2015). Evidence for horizontal transfer of mitochondrial DNA to the plastid genome in a bamboo genus. Sci Rep..

[16] Rabah SO, Lee C, Hajrah NH, Makki RM, Alharby HF, Alhebshi AM, et al. Plastome sequencing of ten nonmodel crop species uncovers a large insertion of mitochondrial DNA in cashew. Plant Genome. 2017;10(3). 10.3835/plantgenome2017.03.0020 29293812

[17] Raman G, Park S, Lee EM, Park S (2019). Evidence of mitochondrial DNA in the chloroplast genome of *Convallaria keiskei* and its subsequent evolution in the Asparagales. Sci Rep..

[18] Wu CS, Chen CI, Chaw SM (2022). Plastid phylogenomics and plastome evolution in the morning glory family (Convolvulaceae). Front Plant Sci..

[19] Koulintchenko M, Konstantinov Y, Dietrich A (2003). Plant mitochondria actively import DNA via the permeability transition pore complex. EMBO J..

[20] Arimura S, Yamamoto J, Aida GP, Nakazono M, Tsutsumi N (2004). Frequent fusion and fission of plant mitochondria with unequal nucleoid distribution. Proc Natl Acad Sci U S A..

[21] Mower JP, Jain K, Hepburn NJ. The role of horizontal transfer in shaping the plant mitochondrial genome. Maréchal-Drouard L, editor. Advances in botanical research. New York: Academic Press; 2012. p. 41–69.

[22] Sanchez-Puerta MV, Edera A, Gandini CL, Williams AV, Howell KA, Nevill PG (2019). Genome-scale transfer of mitochondrial DNA from legume hosts to the holoparasite *Lophophytum mirabile* (Balanophoraceae). Mol Phylogenet Evol..

[23] Garcia LE, Edera AA, Palmer JD, Sato H, Sanchez-Puerta MV (2021). Horizontal gene transfers dominate the functional mitochondrial gene space of a holoparasitic plant. New Phytol..

[24] Davis CC, Anderson WR, Wurdack KJ (2005). Gene transfer from a parasitic flowering plant to a fern. Proc Biol Sci..

[25] Petersen G, Anderson B, Braun HP, Meyer EH, Møller IM (2020). Mitochondria in parasitic plants. Mitochondrion..

[26] Won H, Renner SS. The internal transcribed spacer of nuclear ribosomal DNA in the gymnosperm *Gnetum*. Mol Phylogenet Evol. 2005;36(3):581–97. 10.1016/j.ympev.2005.03.011 16099382

[27] Price RA (1996). Systematics of the Gnetales: a review of morphological and molecular evidence. Int. J. Plant Sci..

[28] Feild TS, Balun L (2008). Xylem hydraulic and photosynthetic function of *Gnetum* (Gnetales) species from Papua New Guinea. New Phytol..

[29] Deng N, Hou C, Liu C, Li M, Bartish I, Tian Y (2019). Significance of photosynthetic characters in the evolution of Asian *Gnetum* (Gnetales). Front Plant Sci..

[30] Hou C, Humphreys AM, Thureborn O, Rydin C (2015). New insights into the evolutionary history of *Gnetum* (Gnetales). Taxon..

[31] Won H, Renner SS (2006). Dating dispersal and radiation in the gymnosperm *Gnetum* (Gnetales)—clock calibration when outgroup relationships are uncertain. Syst Biol..

[32] Antipov D, Korobeynikov A, McLean JS, Pevzner PA (2016). hybridSPAdes: an algorithm for hybrid assembly of short and long reads. Bioinformatics..

[33] Gandini CL, Sanchez-Puerta MV (2017). Foreign plastid sequences in plant mitochondria are frequently acquired via mitochondrion-to-mitochondrion horizontal transfer. Sci Rep..

[34] Liu H, Zhao W, Zhang RG, Mao JF, Wang XR (2022). Repetitive elements, sequence turnover and cyto-nuclear gene transfer in gymnosperm mitogenomes. Front Genet..

[35] Wu CS, Chaw SM (2022). Evolution of mitochondrial RNA editing in extant gymnosperms. Plant J..

[36] Rydin C, Wikström N, Bremer B (2017). Conflicting results from mitochondrial genomic data challenge current views of Rubiaceae phylogeny. Am J Bot..

[37] Magallón S, Castillo A (2009). Angiosperm diversification through time. Am J Bot..

[38] Putintseva YA, Bondar EI, Simonov EP, Sharov VV, Oreshkova NV, Kuzmin DA, et al. Siberian larch (*Larix sibirica* Ledeb.) mitochondrial genome assembled using both short and long nucleotide sequence reads is currently the largest known mitogenome. BMC Genomics. 2020;21(1):654. 10.1186/s12864-020-07061-4 PMC7517811 32972367

[39] Gurdon C, Svab Z, Feng Y, Kumar D, Maliga P (2016). Cell-to-cell movement of mitochondria in plants. Proc Natl Acad Sci U S A..

[40] Hertle AP, Haberl B, Bock R. Horizontal genome transfer by cell-to-cell travel of whole organelles. Sci Adv. 2021;7(1):eabd8215. 10.1126/sciadv.abd8215 PMC7775762 33523859

[41] Huang J (2013). Horizontal gene transfer in eukaryotes: the weak-link model. Bioessays..

[42] Stewart CNJr, Via LE. A rapid CTAB DNA isolation technique useful for RAPD fingerprinting and other PCR applications. Biotechniques. 1993;14(5):748–50. 8512694

[43] Wick RR, Judd LM, Gorrie CL, Holt KE (2017). Unicycler: Resolving bacterial genome assemblies from short and long sequencing reads. PLoS Comput Biol..

[44] Wu CS, Sudianto E, Chiu HL, Chao CP, Chaw SM (2021). Reassessing banana phylogeny and organelle inheritance modes using genome skimming data. Front Plant Sci..

[45] Bankevich A, Nurk S, Antipov D, Gurevich AA, Dvorkin M, Kulikov AS (2012). SPAdes: a new genome assembly algorithm and its applications to single-cell sequencing. J Comput Biol..

[46] Walker BJ, Abeel T, Shea T, Priest M, Abouelliel A, Sakthikumar S (2014). Pilon: an integrated tool for comprehensive microbial variant detection and genome assembly improvement. PLoS One..

[47] Geneious Prime. https://www.geneious.com.

[48] Chan PP, Lin BY, Mak AJ, Lowe TM. tRNAscan-SE 2.0: improved detection and functional classification of transfer RNA genes. Nucleic Acids Res. 2021;49(16):9077‒96. 10.1093/nar/gkab688 PMC8450103 34417604

[49] Quinlan AR, Hall IM (2010). BEDTools: a flexible suite of utilities for comparing genomic features. Bioinformatics..

[50] Kurtz S, Phillippy A, Delcher AL, Smoot M, Shumway M, Antonescu C (2004). Versatile and open software for comparing large genomes. Genome Biol..

[51] Langmead B, Salzberg SL (2012). Fast gapped-read alignment with Bowtie 2. Nat Methods..

[52] Kim D, Pertea G, Trapnell C, Pimentel H, Kelley R, Salzberg SL (2013). TopHat2: accurate alignment of transcriptomes in the presence of insertions, deletions and gene fusions. Genome Biol..

[53] Li H, Handsaker B, Wysoker A, Fennell T, Ruan J, Homer N (2009). The sequence alignment/map format and SAMtools. Bioinformatics..

[54] Lenz H, Hein A, Knoop V. Plant organelle RNA editing and its specificity factors: enhancements of analyses and new database features in PREPACT 3.0. BMC Bioinformatics. 2018;19(1):255. 10.1186/s12859-018-2244-9 PMC6029061 29970001

[55] Katoh K, Standley DM (2013). MAFFT multiple sequence alignment software version 7: improvements in performance and usability. Mol Biol Evol..

[56] Minh BQ, Schmidt HA, Chernomor O, Schrempf D, Woodhams MD, von Haeseler A (2020). IQ-TREE 2: new models and efficient methods for phylogenetic inference in the genomic era. Mol Biol Evol..

[57] Rambaut A. Figtree v1.4.4. http://tree.bio.ed.ac.uk/software/figtree/ (2018)

[58] Krzywinski M, Schein J, Birol I, Connors J, Gascoyne R, Horsman D (2009). Circos: an information aesthetic for comparative genomics. Genome Res..

[59] Wu CS, Wang RJ, Chaw SM. Horizontal gene transfer from angiosperms to *Gnetum*. https://www.ncbi.nlm.nih.gov/sra/?term=PRJNA1029351 (2024).

[60] Wu CS, Wang RJ, Chaw SM. Asian *Gnetum* mitogenomes. https://www.ncbi.nlm.nih.gov/sra/?term=PRJNA1029324 (2024). 10.1186/s12915-024-01924-y PMC11197197 38915079

[61] Wu CS, Wang RJ, Chaw SM. Asian *Gnetum* transcriptomes. https://www.ncbi.nlm.nih.gov/sra/?term=PRJNA1029334 (2024).

[62] Wu CS, Chaw SM. Asian *Gnetum* mitogenomes. GenBank. https://www.ncbi.nlm.nih.gov/nuccore/?term=LC783565%3ALC783658%5Baccn%5D (2024). 10.1186/s12915-024-01924-y PMC11197197 38915079

