# Supplementary material for: Integration of large and diverse angiosperm DNA fragments into Asian Gnetum mitogenomes
Source: BMC Biol. 2024 Jun 25;22:140. doi: 10.1186/s12915-024-01924-y (PMC11197197; doi:10.1186/s12915-024-01924-y)
Supplement: Supplementary file 1 — Additional file 1: Figs S1‒S24. Fig. S1. The mitogenome map of Gnetum gnemon. Grey bars represent nine circular-mapping chromosomes that are displayed as linear molecules for easy comparisons. Loci are color-coded depending on their origins. Light-blue histograms denote DNA read depths in log scales. Chr, chromosome. Fig. S2. The draft mitogenome map of Gnetum ula. Grey bars represent 21 linear scaffolds. Loci are color-coded depending on their origins. Light-blue histograms denote DNA read depths in log scales. SC, scaffold. Fig. S3. A ML tree inferred from ccmB using ferns as the outgroup. Subtrees within left boxes indicate the relative placements of foreign and native ccmB genes in Gnetum with bootstrap values under a 50% majority rule. Fig. S4. A ML tree inferred from ccmFc using ferns as the outgroup. Subtrees within left boxes indicate the relative placements of foreign and native ccmFc genes in Gnetum with bootstrap values under a 50% majority rule. Fig. S5. A ML tree inferred from ccmFn using ferns as the outgroup. Subtrees within left boxes indicate the relative placements of foreign and native ccmFn genes in Gnetum with bootstrap values under a 50% majority rule. Fig. S6. A ML tree inferred from matR using ferns as the outgroup. Subtrees within left boxes indicate the relative placements of foreign and native matR genes in Gnetum with bootstrap values under a 50% majority rule. Fig. S7. A ML tree inferred from nad1 exons 2‒3 using ferns as the outgroup. Subtrees within left boxes indicate the relative placements of foreign and native nad1 exons 2‒3 loci in Gnetum with bootstrap values under a 50% majority rule. Fig. S8. A ML tree inferred from nad1 exons 4‒5 using ferns as the outgroup. Subtrees within left boxes indicate the relative placements of foreign and native nad1 exons 4‒5 loci in Gnetum with bootstrap values under a 50% majority rule. Fig. S9. A ML tree inferred from nad5 exons 4‒5 using ferns as the outgroup. Subtrees within left boxes indicate [file 12915_2024_1924_MOESM1_ESM.pdf]

Supplementary figures for

Integration of large and diverse angiosperm DNA fragments into Asian *Gnetum* mitogenomes

Chung-Shien Wu[1], Rui-Jiang Wang[2], Shu-Miaw Chaw[1*]

[1]Biodiversity Research Center, Academia Sinica, Taiwan; 2South China Botanical Garden, Chinese Academy of Science, China.
[*]Correspondence: smchaw@sinica.edu.tw

This file includes: Figures S1–S24.

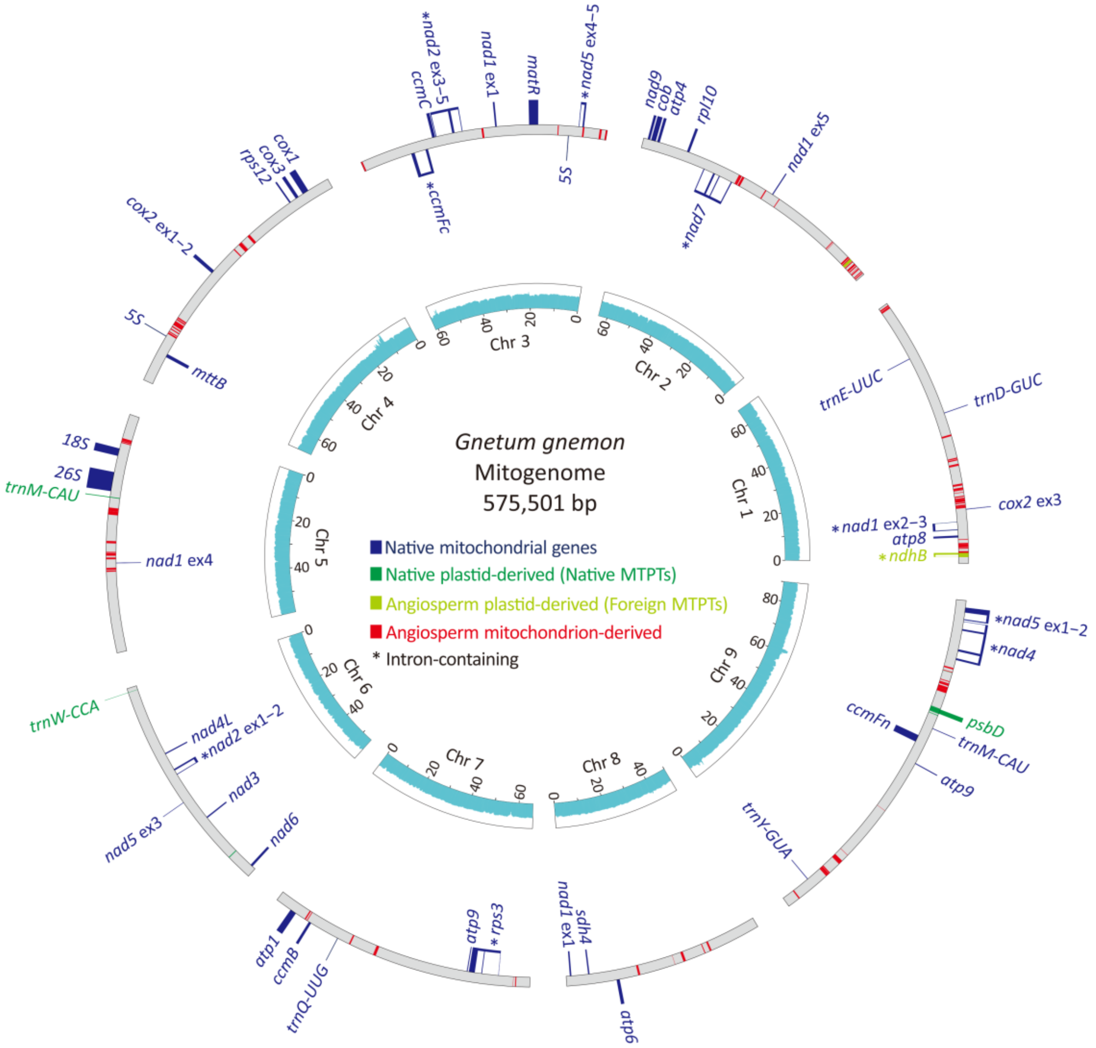

Fig. S1. Mitogenome map of *Gnetum gnemon*. Grey bars represent nine circular-mapping chromosomes that are displayed as linear molecules for easy comparisons. Loci are color-coded depending on their origins. Light-blue histograms denote DNA read depths in log scale. Chr, chromosome.

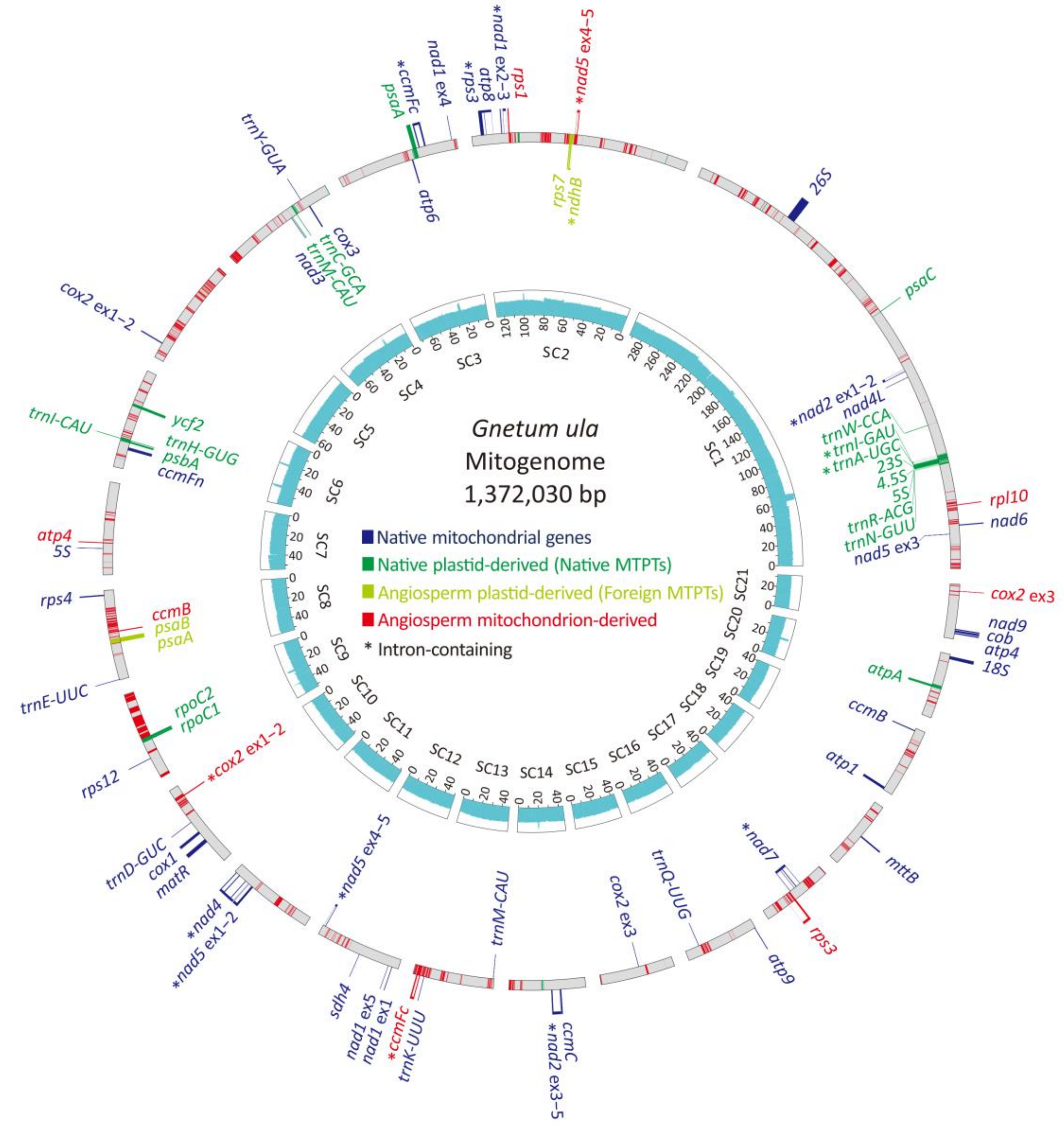

Fig. S2. Draft mitogenome map of *Gnetum ula*. Grey bars represent 21 linear scaffolds. Loci are color-coded depending on their origins. Light-blue histograms denote DNA read depths in log scale. SC, scaffold.

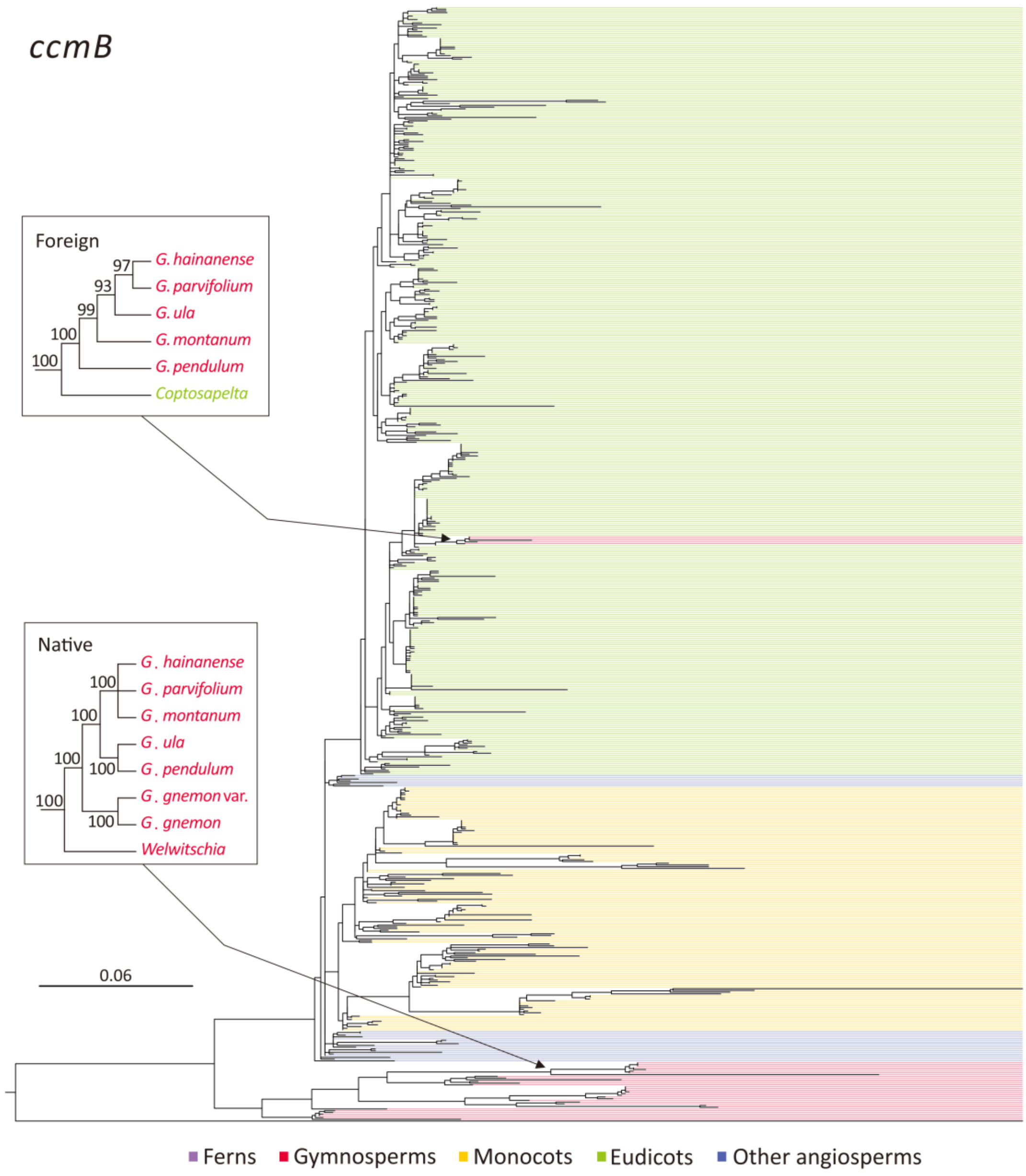

Fig. S3. A ML tree inferred from *ccmB* using ferns as the outgroup. Subtrees within left boxes indicate the relative placements of foreign and native *ccmB* genes in *Gnetum* with bootstrap values under a 50% majority rule.

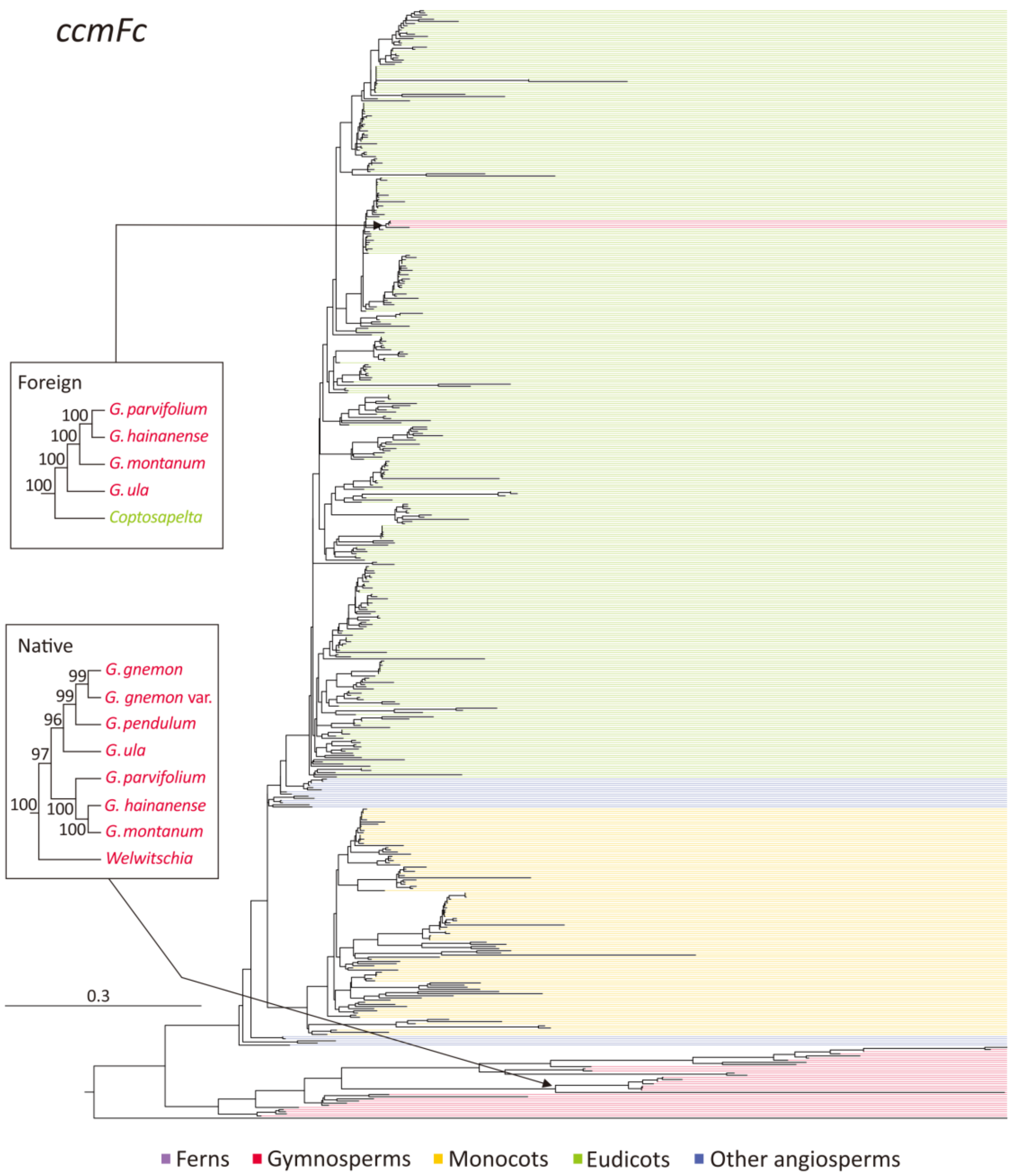

Fig. S4. A ML tree inferred from *ccmFc* using ferns as the outgroup. Subtrees within left boxes indicate the relative placements of foreign and native *ccmFc* genes in *Gnetum* with bootstrap values under a 50% majority rule.

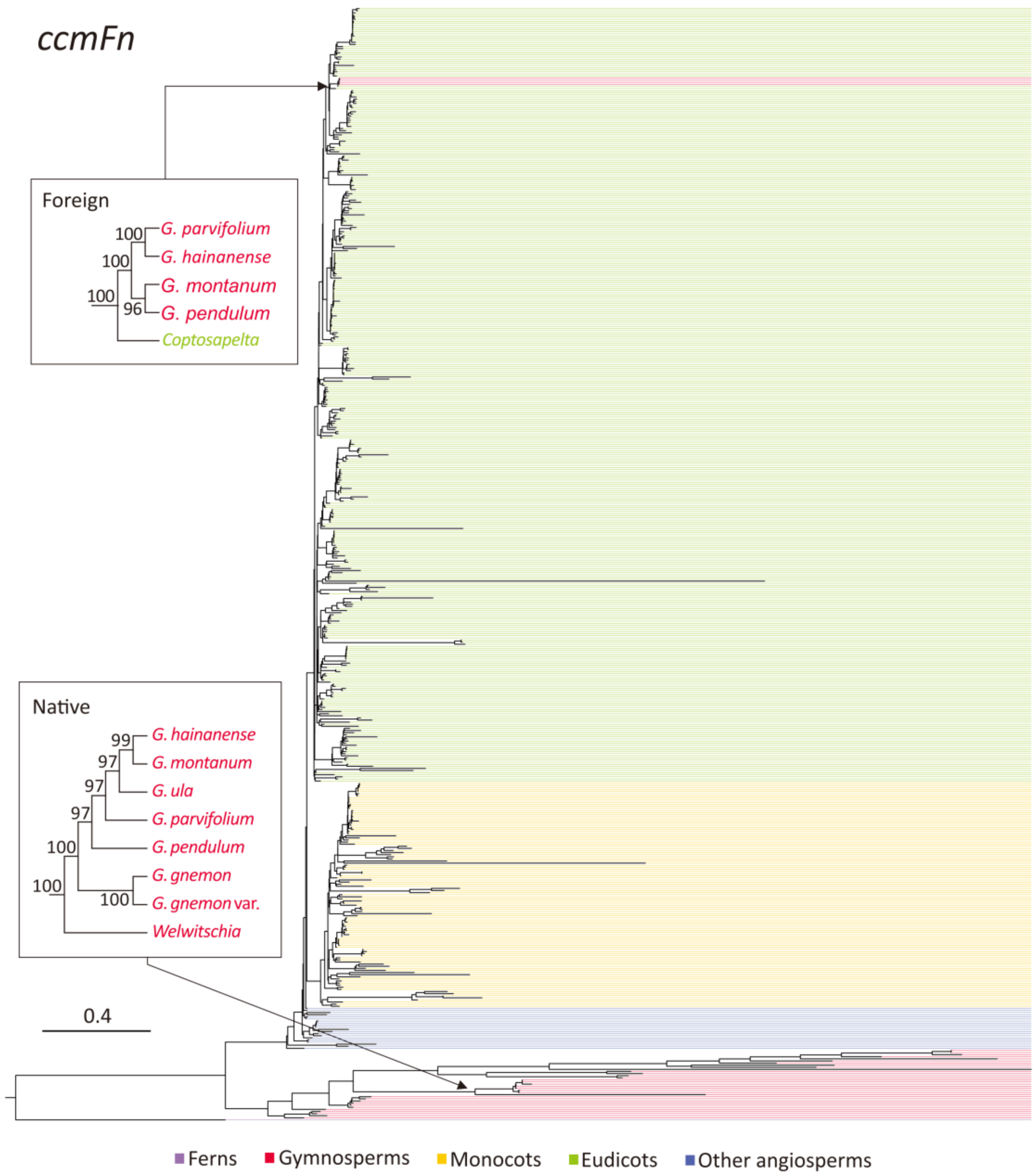

Fig. S5. A ML tree inferred from *ccmFn* using ferns as the outgroup. Subtrees within left boxes indicate the relative placements of foreign and native *ccmFn* genes in *Gnetum* with bootstrap values under a 50% majority rule.

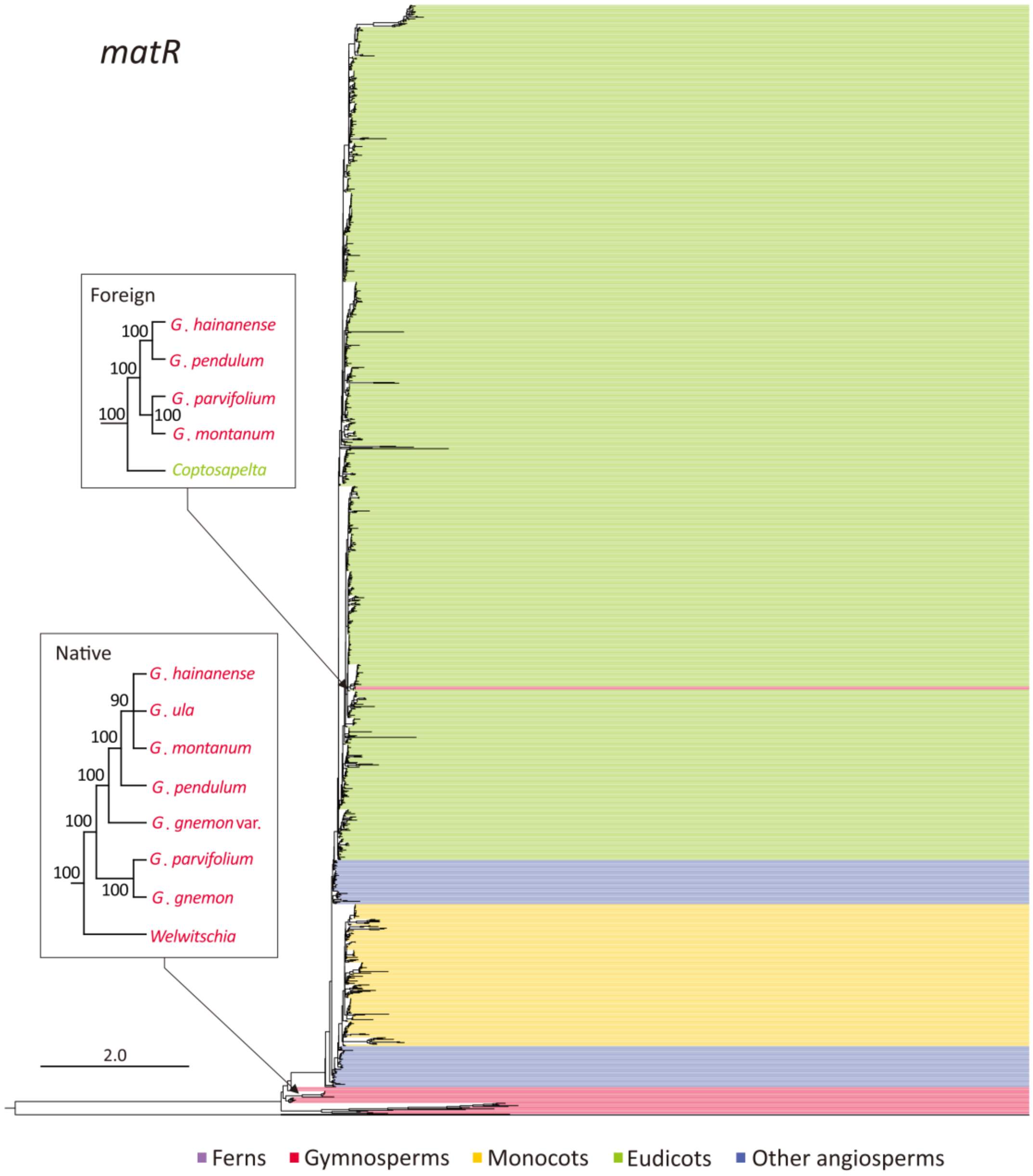

Fig. S6. A ML tree inferred from *matR* using ferns as the outgroup. Subtrees within left boxes indicate the relative placements of foreign and native *matR* genes in *Gnetum* with bootstrap values under a 50% majority rule.

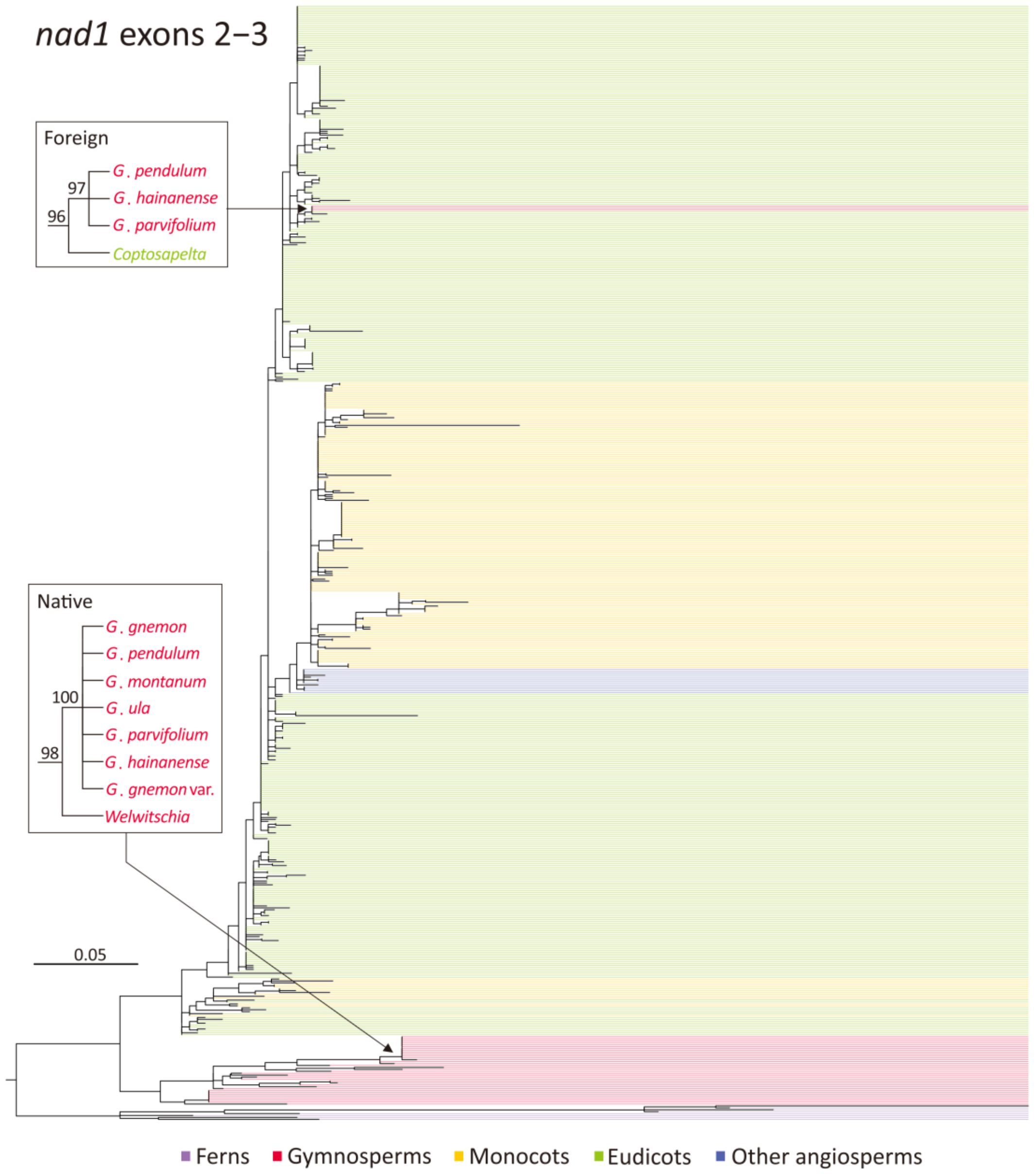

Fig. S7. A ML tree inferred from *nad1* exons 2–3 using ferns as the outgroup. Subtrees within left boxes indicate the relative placements of foreign and native *nad1* exons 2–3 loci in *Gnetum* with bootstrap values under a 50% majority rule.

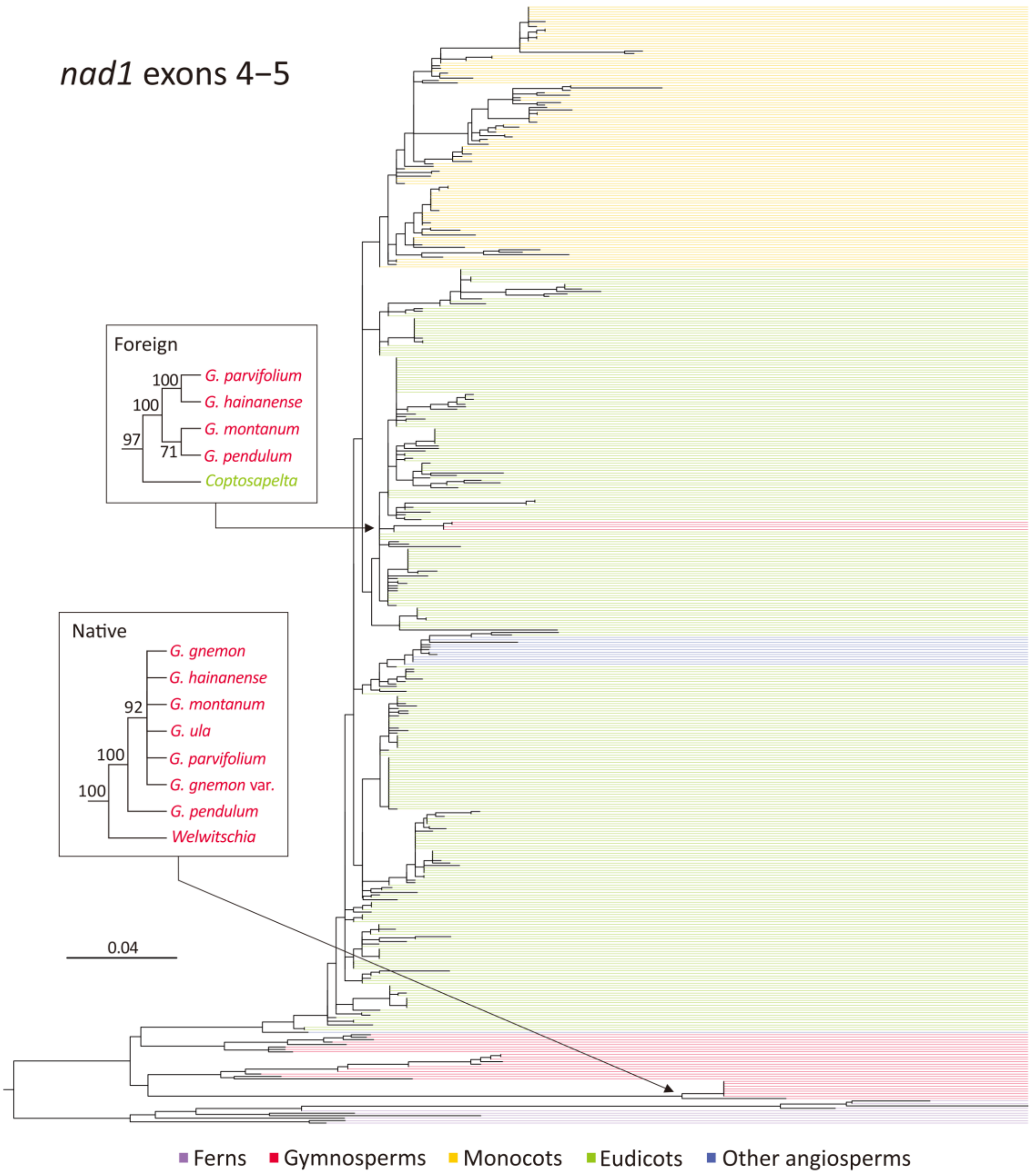

Fig. S8. A ML tree inferred from *nad1* exons 4–5 using ferns as the outgroup. Subtrees within left boxes indicate the relative placements of foreign and native *nad1* exons 4–5 loci in *Gnetum* with bootstrap values under a 50% majority rule.

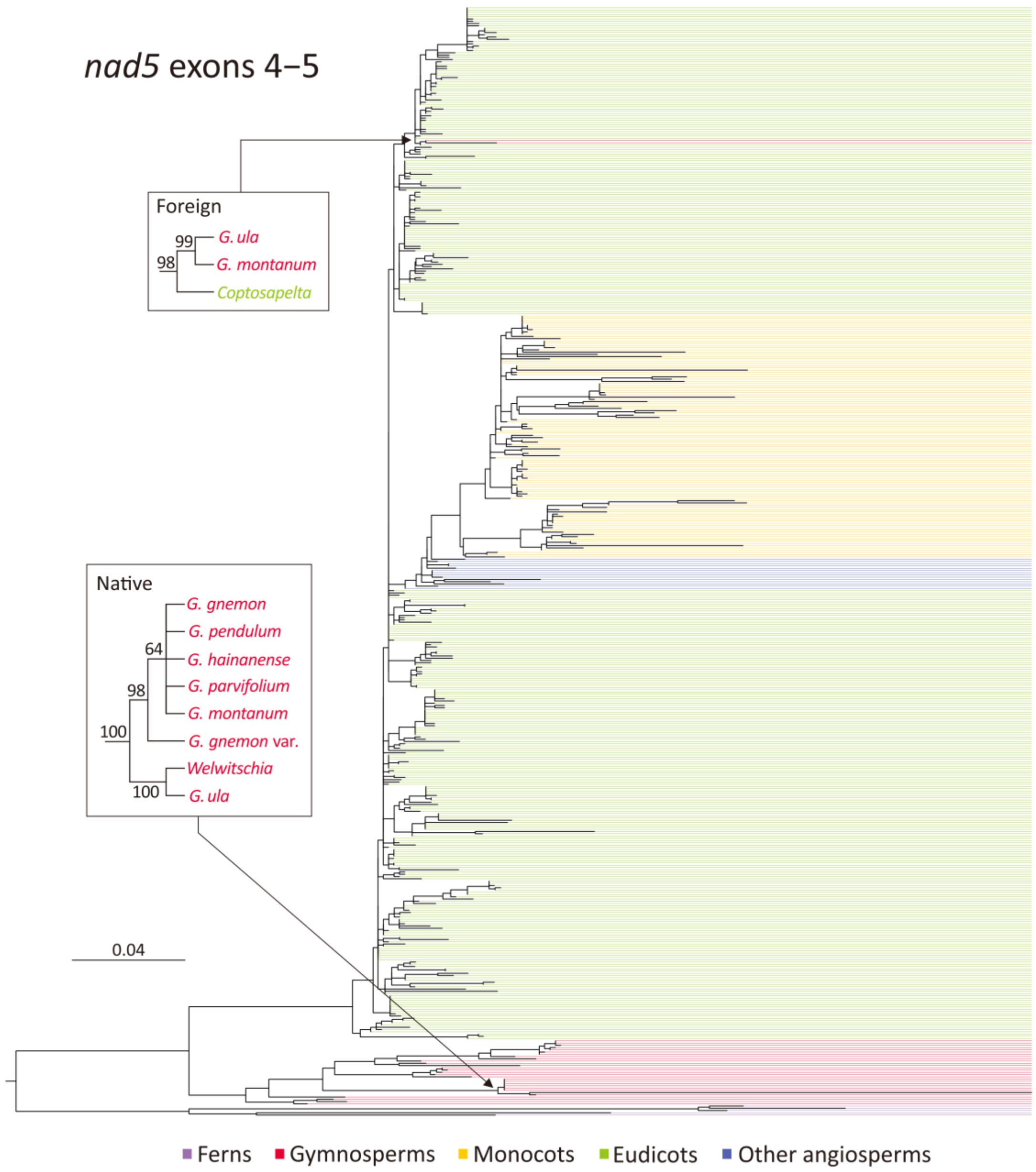

Fig. S9. A ML tree inferred from *nad5* exons 4–5 using ferns as the outgroup. Subtrees within left boxes indicate the relative placements of foreign and native *nad5* exons 4–5 loci in *Gnetum* with bootstrap values under a 50% majority rule.

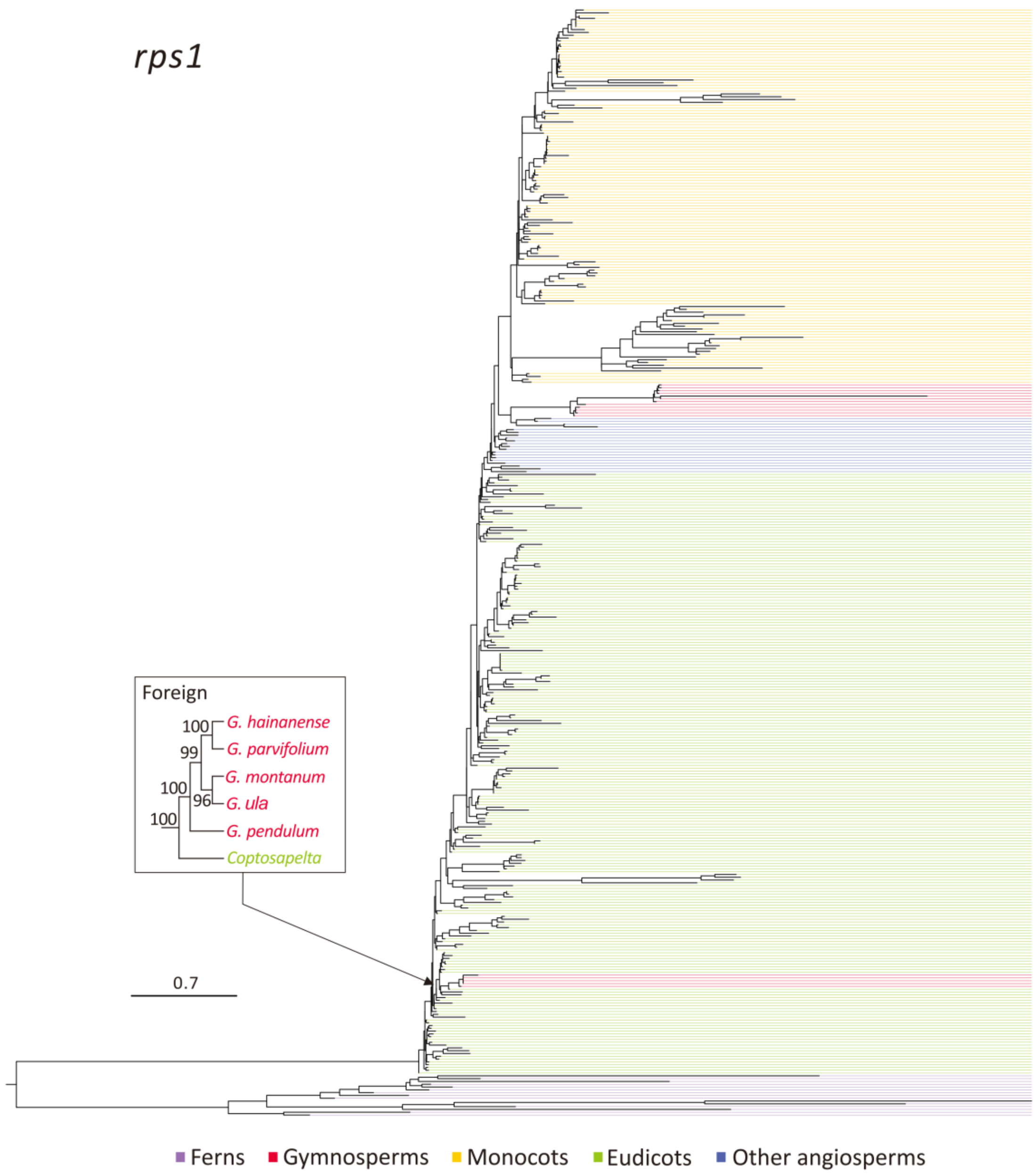

Fig. S10. A ML tree inferred from *rps1* using ferns as the outgroup. The subtree indicates the relative placement of foreign *rps1* genes in *Gnetum* with bootstrap values under a 50% majority rule. The native *rps1* has been lost from *Gnetum* and thus was not included in this tree.

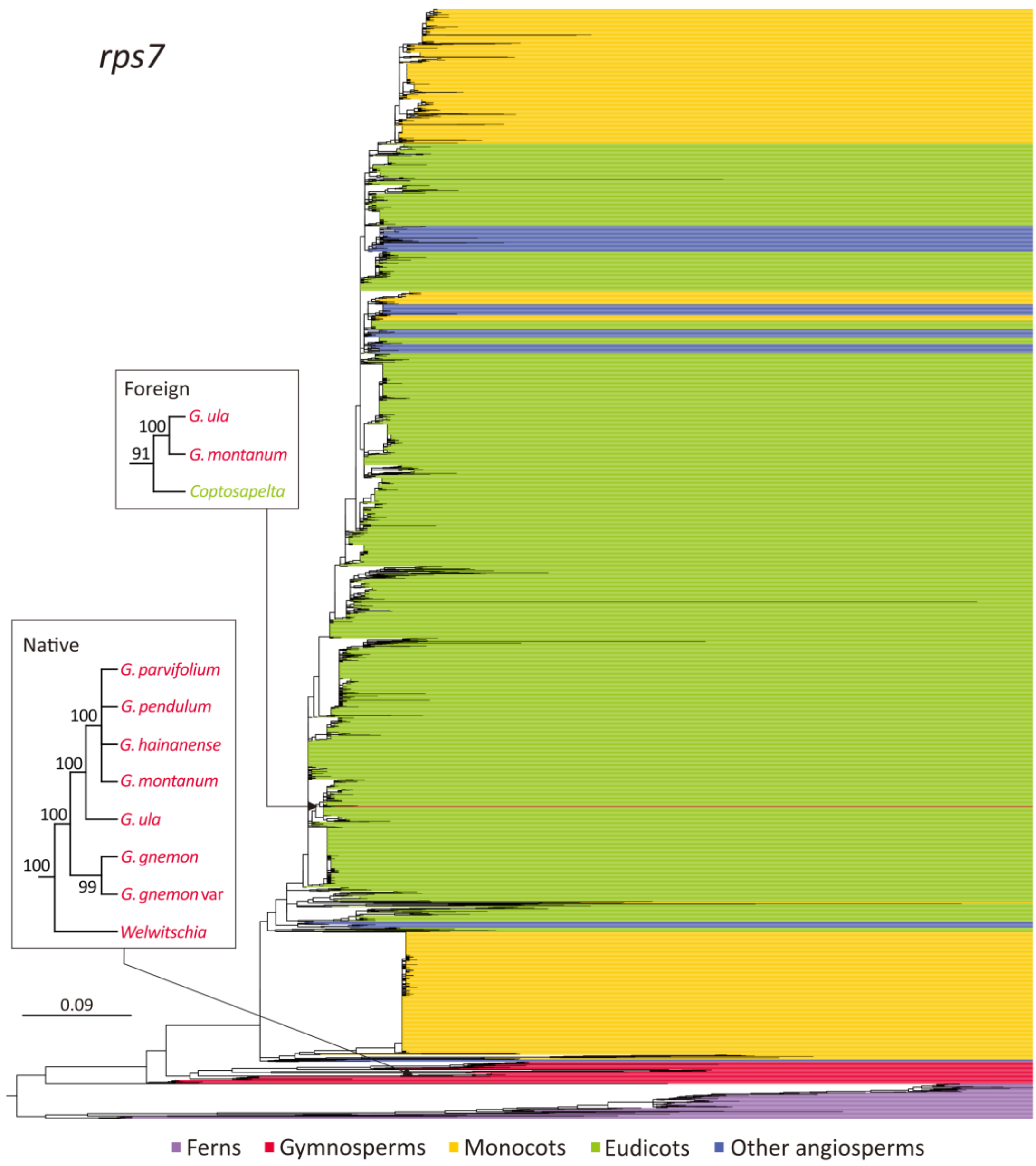

Fig. S11. A ML tree inferred from plastid (native) and mitochondrial plastid-derived (foreign) *rps7* using ferns as the outgroup. Note that *Gnetum*'s plastid loci are separated from their mitochondrial homologs, suggesting that the latter are HGT loci rather than MTPTs. Subtrees detail the relative placements of foreign and native *rps7* genes in *Gnetum* with bootstrap values under a 50% majority rule.

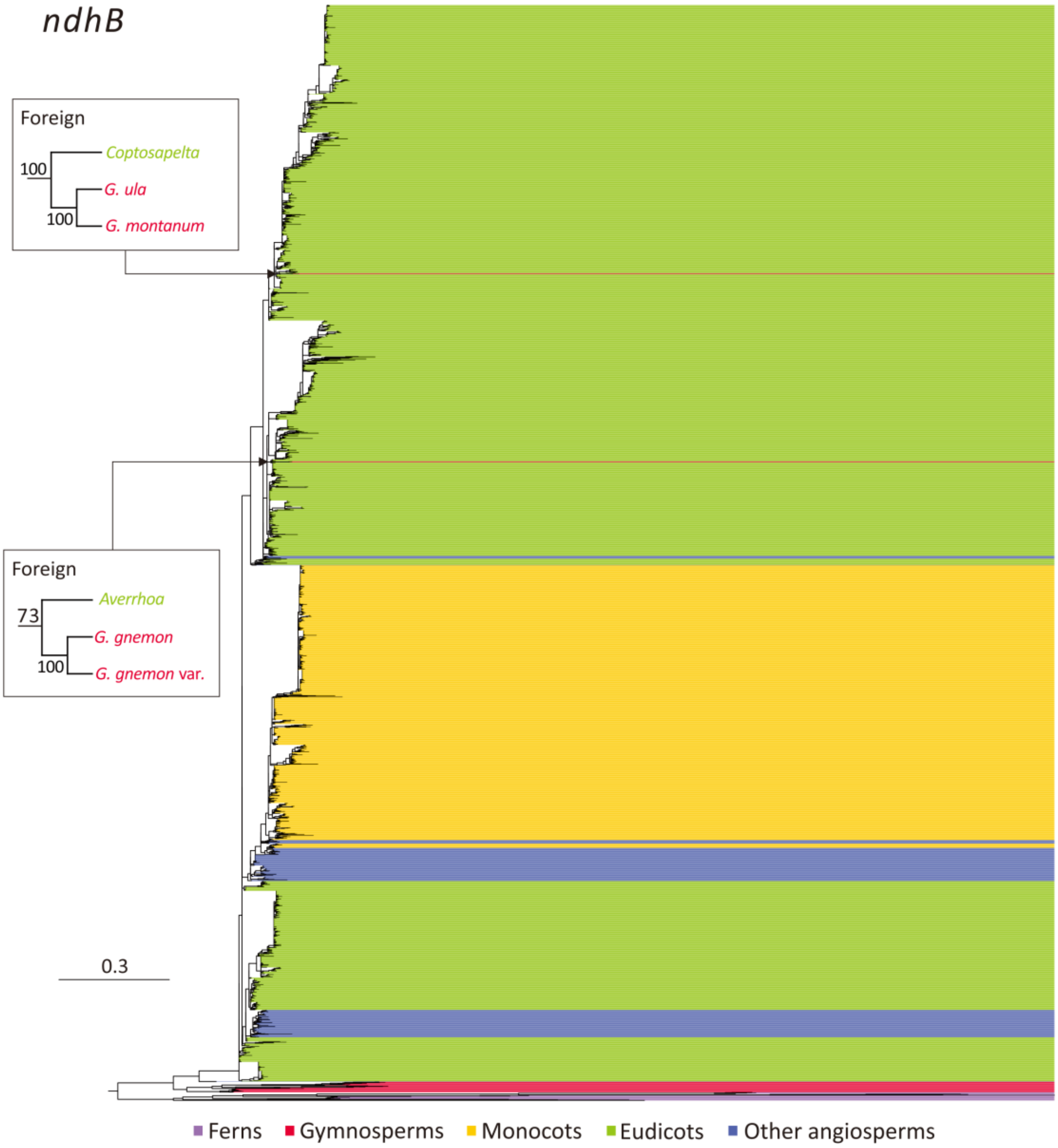

Fig. S12. A ML tree inferred from plastid (native) and mitochondrial plastid-derived (foreign) *ndhB* using ferns as the outgroup. The two remote clades in *Gnetum* suggest that two independent HGT events have taken place in the Asia clades I and II, respectively. Subtrees detail the relative placements of foreign *ndhB* loci in *Gnetum* with bootstrap values under a 50% majority rule. The plastid *ndhB* has been lost from *Gnetum* and thus was not included in this tree.

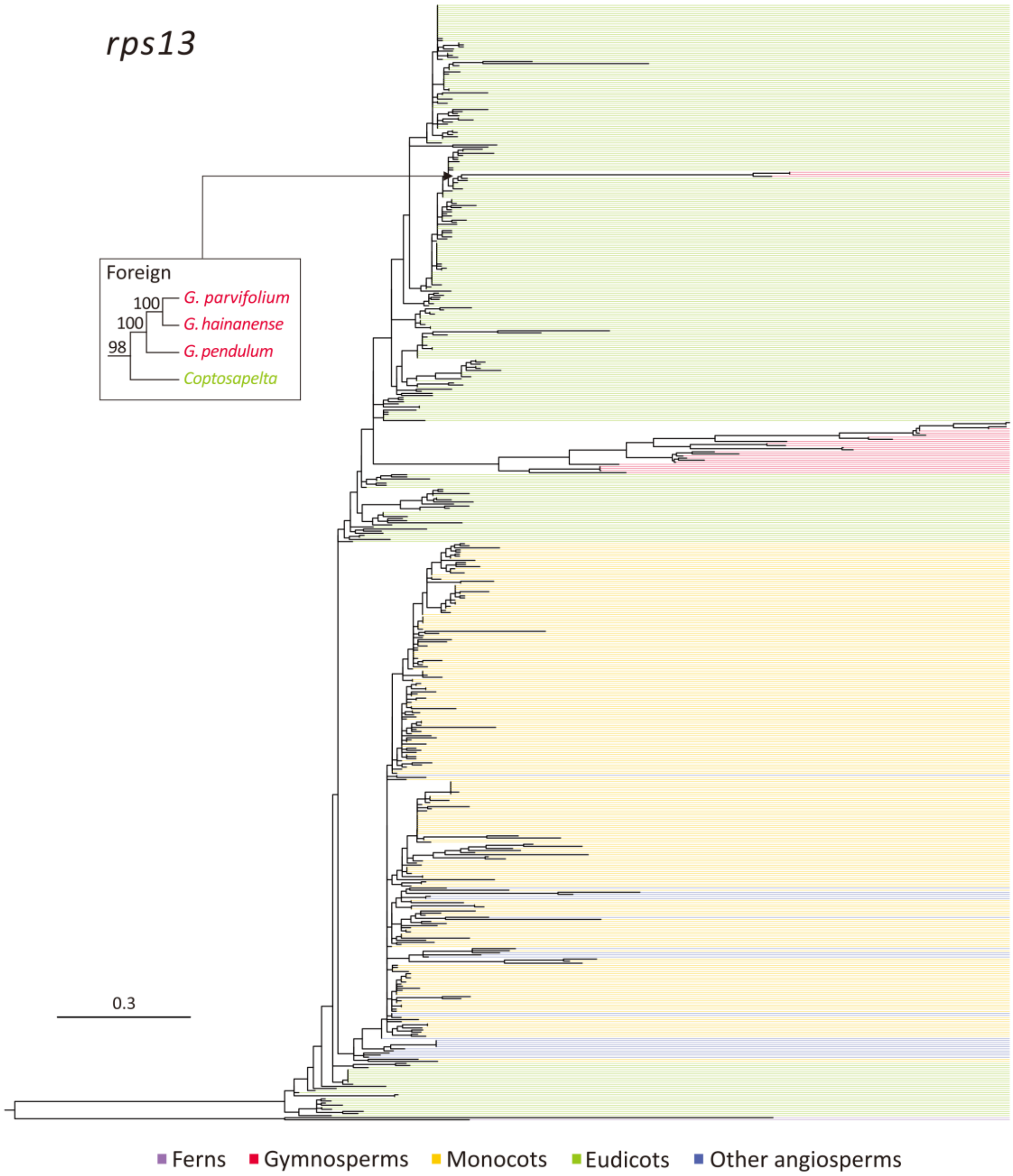

Fig. S13. A ML tree inferred from *rps13* using ferns as the outgroup. The subtree details the relative placements of foreign *rps13* genes in *Gnetum* with bootstrap values under a 50% majority rule. The native *rps13* has been lost from *Gnetum* and thus was not included in this tree.

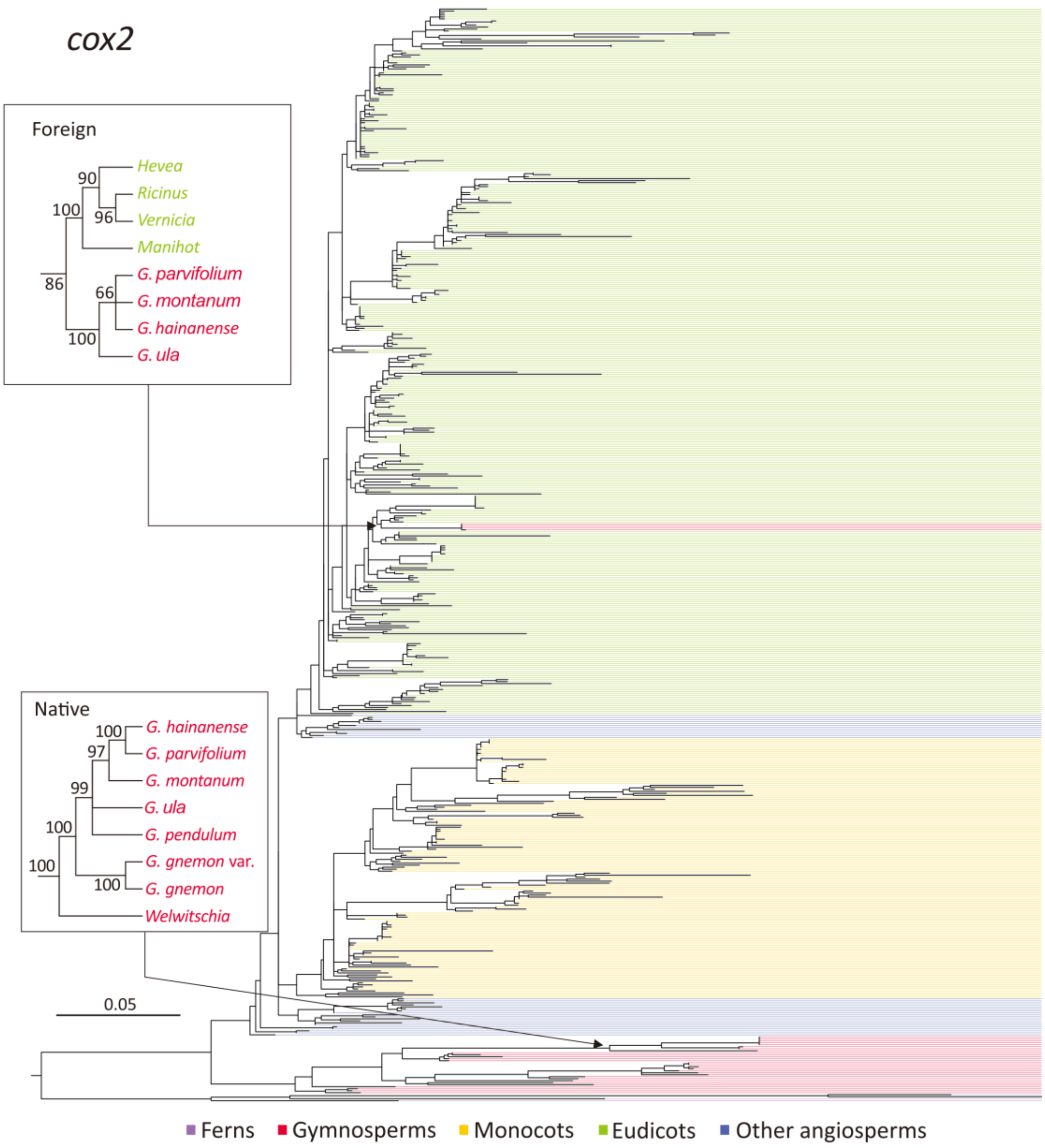

Fig. S14. A ML tree inferred from *cox2* using ferns as the outgroup. Subtrees show the relative placements of foreign and native *cox2* gene in *Gnetum* with bootstrap values under a 50% majority rule.

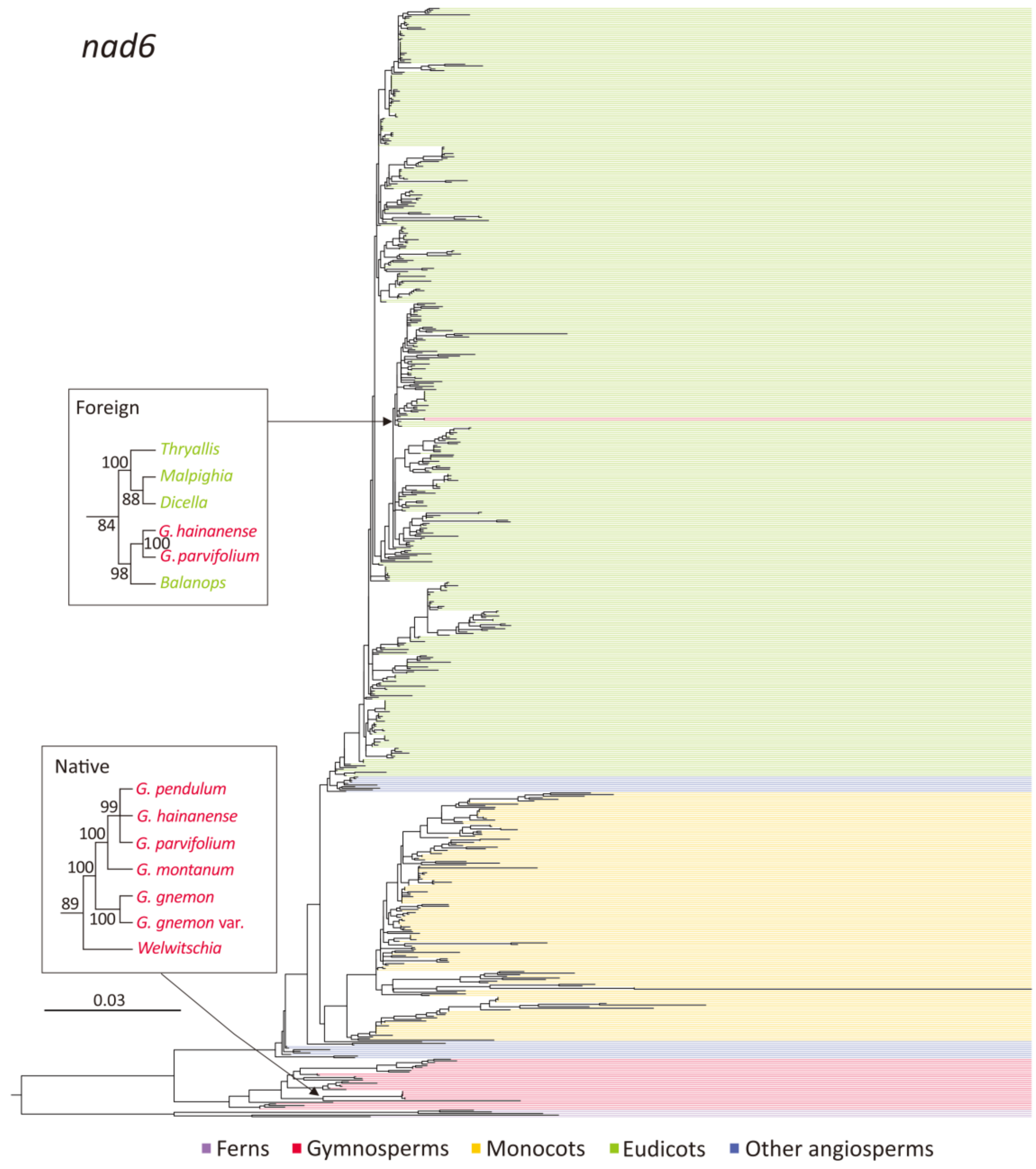

Fig. S15. A ML tree inferred from *nad6* using ferns as the outgroup. Subtrees show the relative placements of foreign and native *nad6* genes in *Gnetum* with bootstrap values under a 50% majority rule.

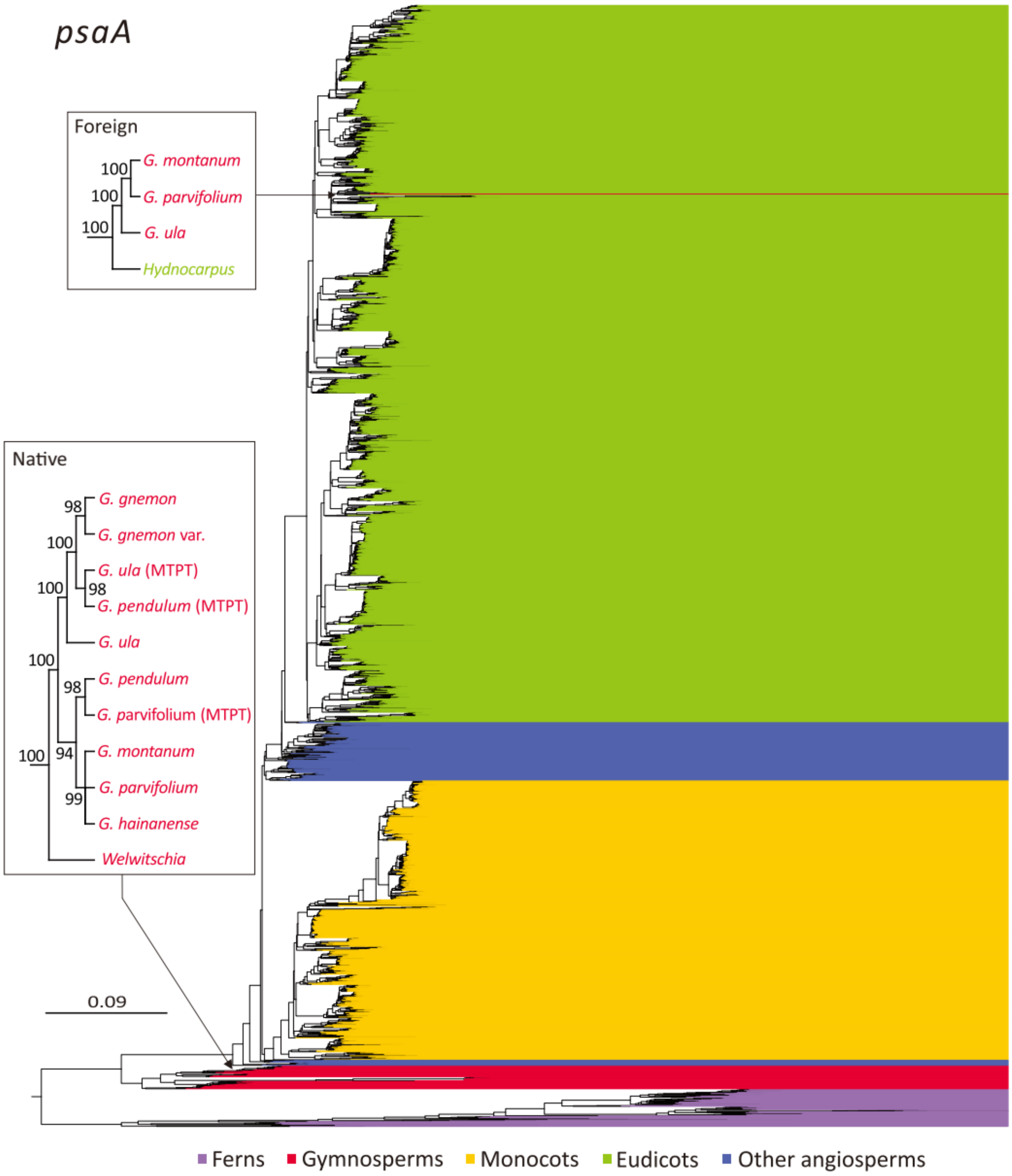

Fig. S16. A ML tree inferred from plastid, native MTPT, and foreign MTPT copies of *psaA* using ferns as the outgroup. Subtrees detail the relative placements of these three *psaA* copies in *Gnetum* with bootstrap values under a 50% majority rule.

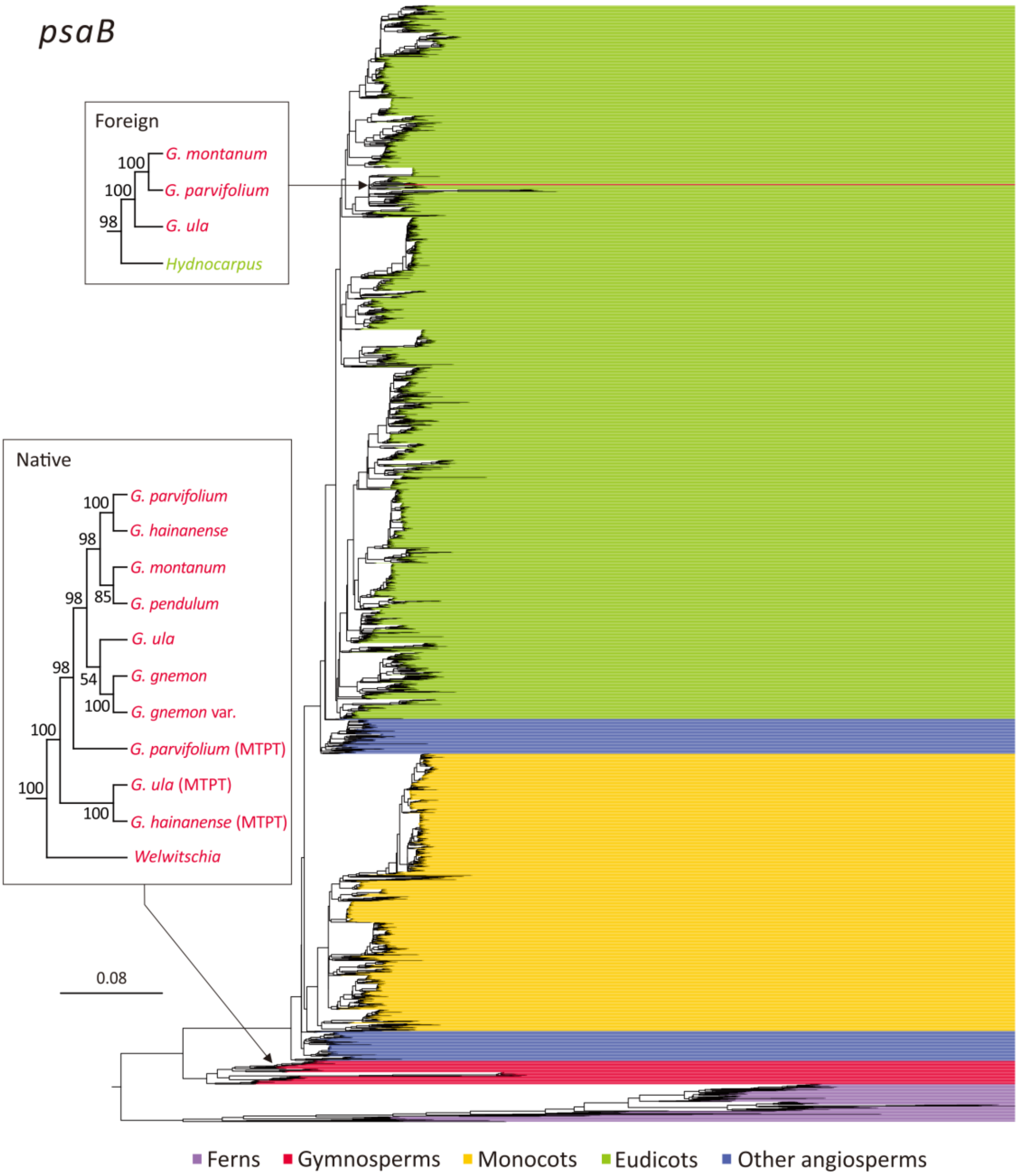

Fig. S17. A ML tree inferred from plastid, native MTPT, and foreign MTPT copies of *psaB* using ferns as the outgroup. Subtrees detail the relative placements of these three *psaB* copies in *Gnetum* with bootstrap values under a 50% majority rule.

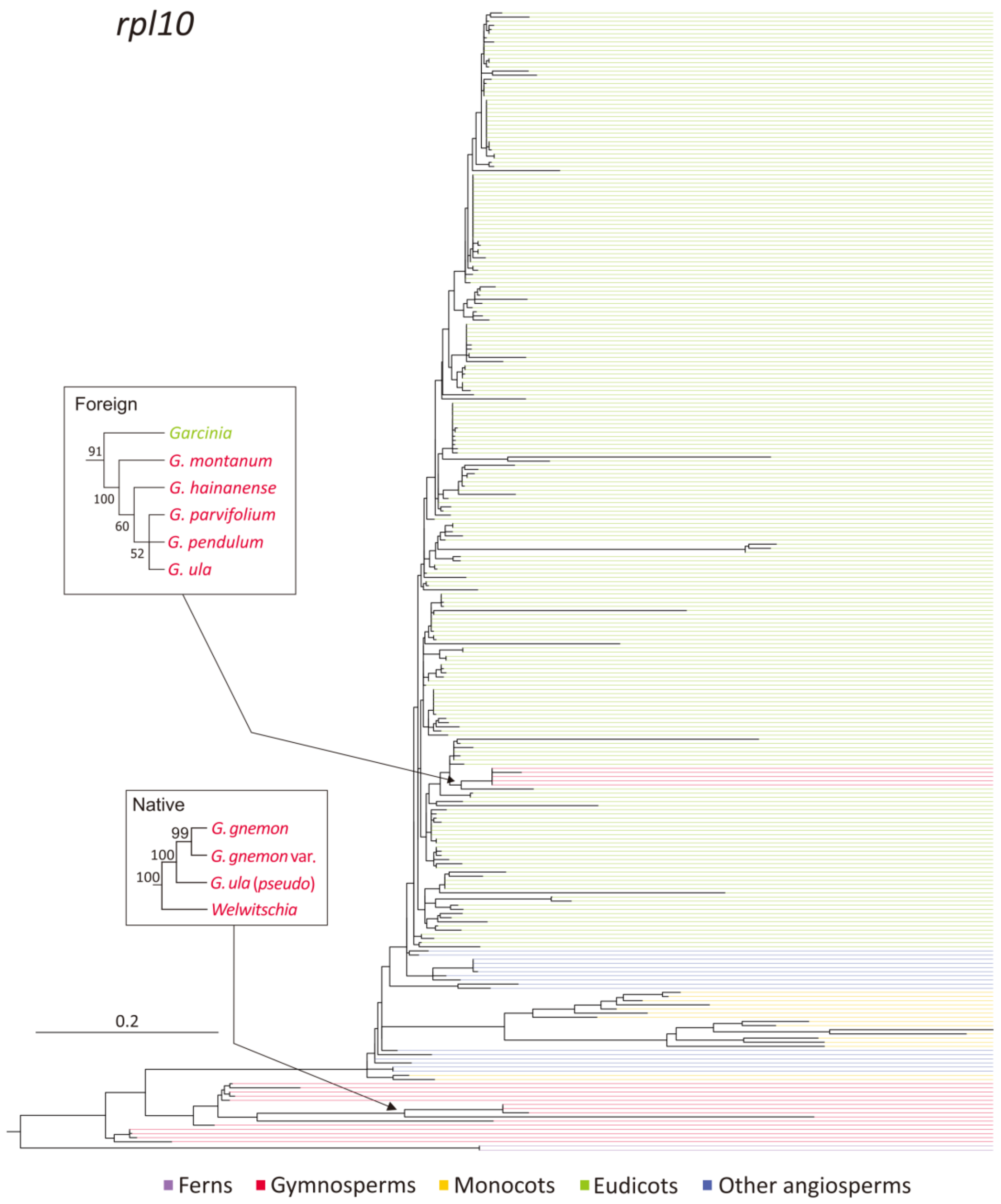

Fig. S18. A ML tree inferred from *rpl10* using ferns as the outgroup. Subtrees detail the relative placements of foreign and native *rpl10* genes in *Gnetum* with bootstrap values under a 50% majority rule. The native *rpl10* has been lost from all sampled *Gnetum* species within the Asia clade II, except for *G. ula* whose *rpl10* is retained but pseudogenized.

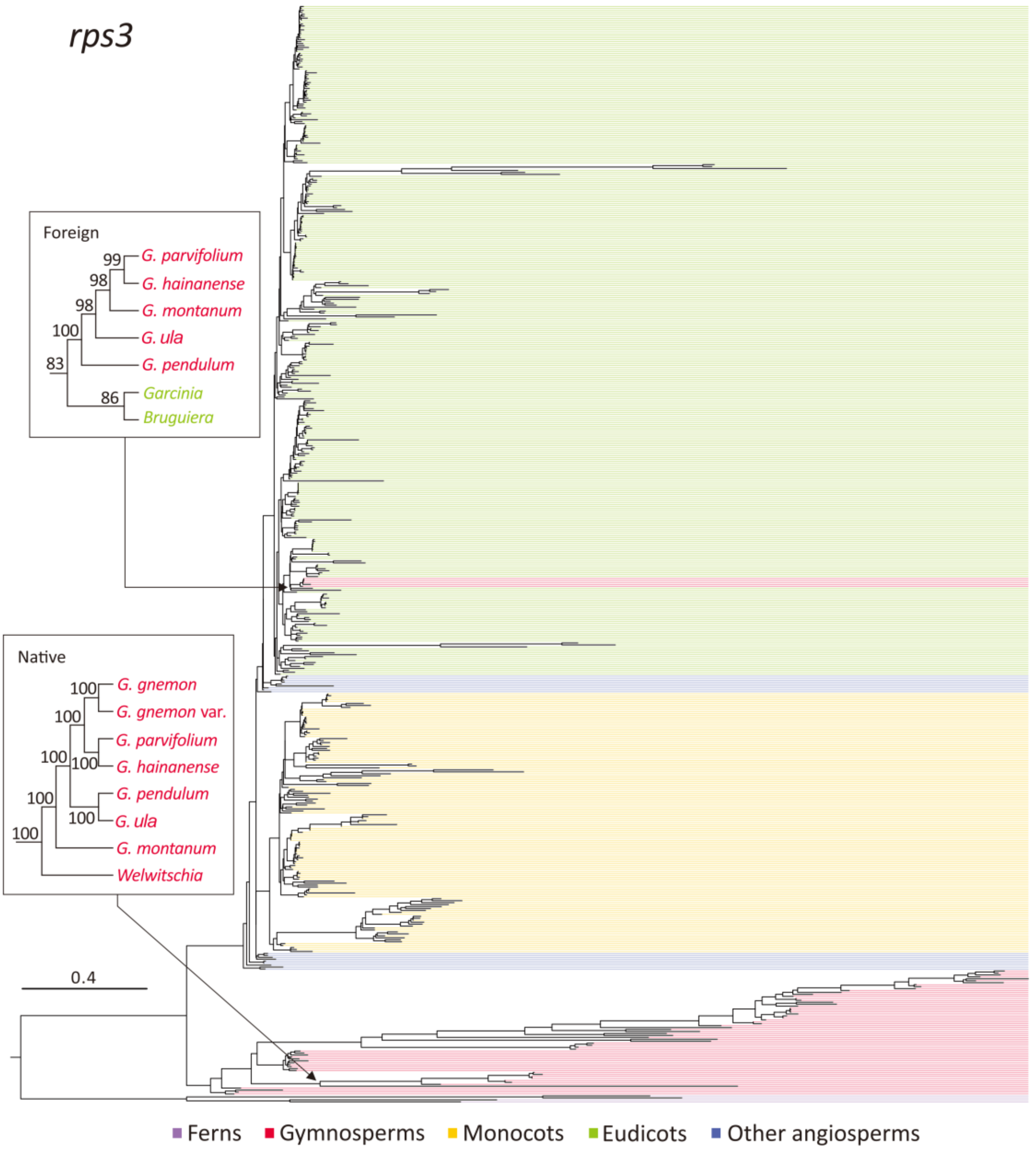

Fig. S19. A ML tree inferred from *rps3* using ferns as the outgroup. Subtrees show the relative placements of foreign and native *rps3* genes in *Gnetum* with bootstrap values under a 50% majority rule.

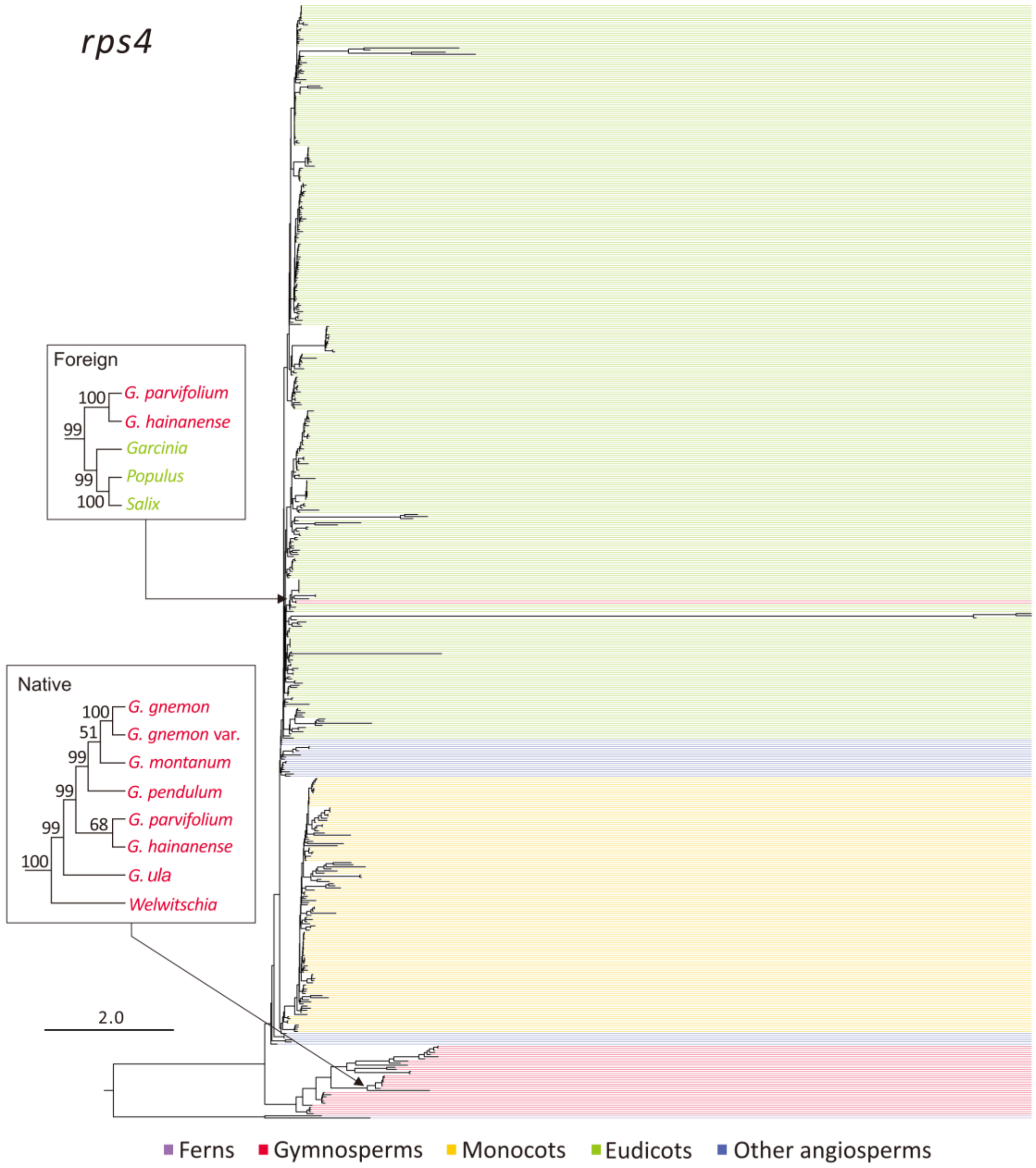

Fig. S20. A ML tree inferred from *rps4* using ferns as the outgroup. Subtrees detail the relative placements of foreign and native *rps4* genes in *Gnetum* with bootstrap values under a 50% majority rule.

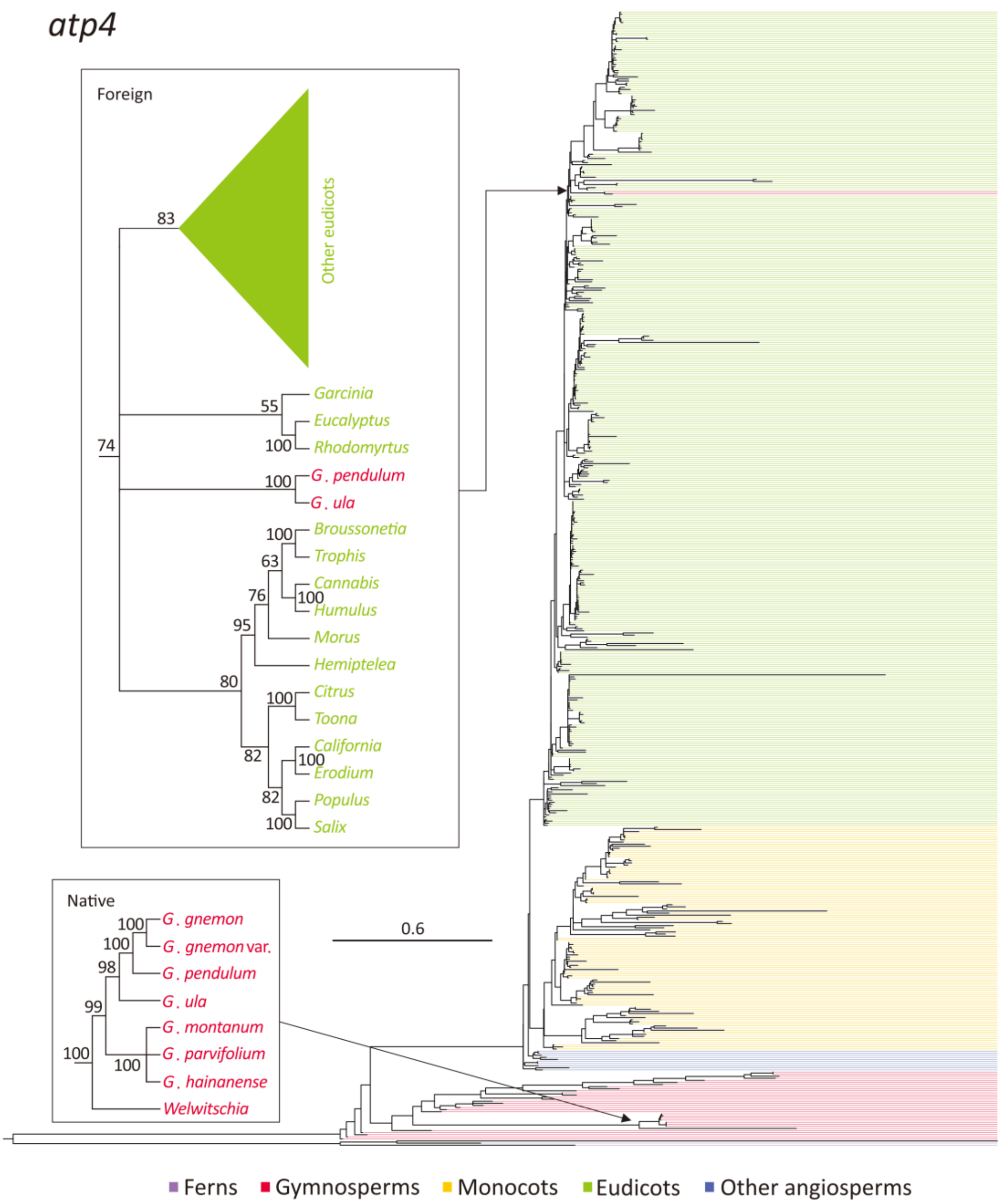

Fig. S21. A ML tree inferred from *atp4* using ferns as the outgroup. Subtrees indicate the relative placements of foreign and native *atp4* in *Gnetum* with bootstrap values under a 50% majority rule. In *Gnetum*, the close relative to foreign *atp4* is uncertain, so that the possible donor of this foreign gene is designated as questionable (“?”) in Figure 2.

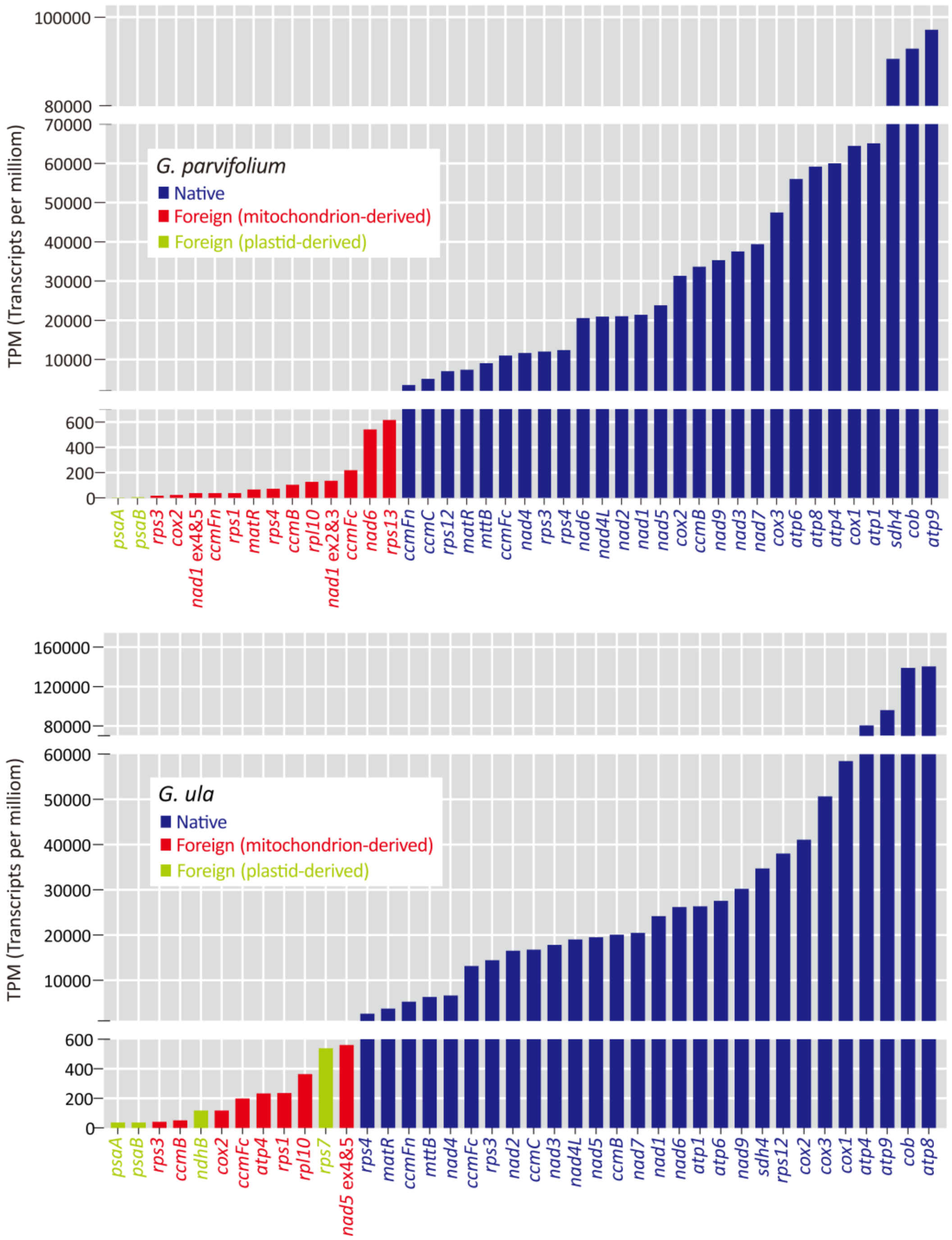

Fig. S22. Comparison of mitochondrial RNA expression level between foreign and native genes in *G. parvifolium* and *G. ula*.

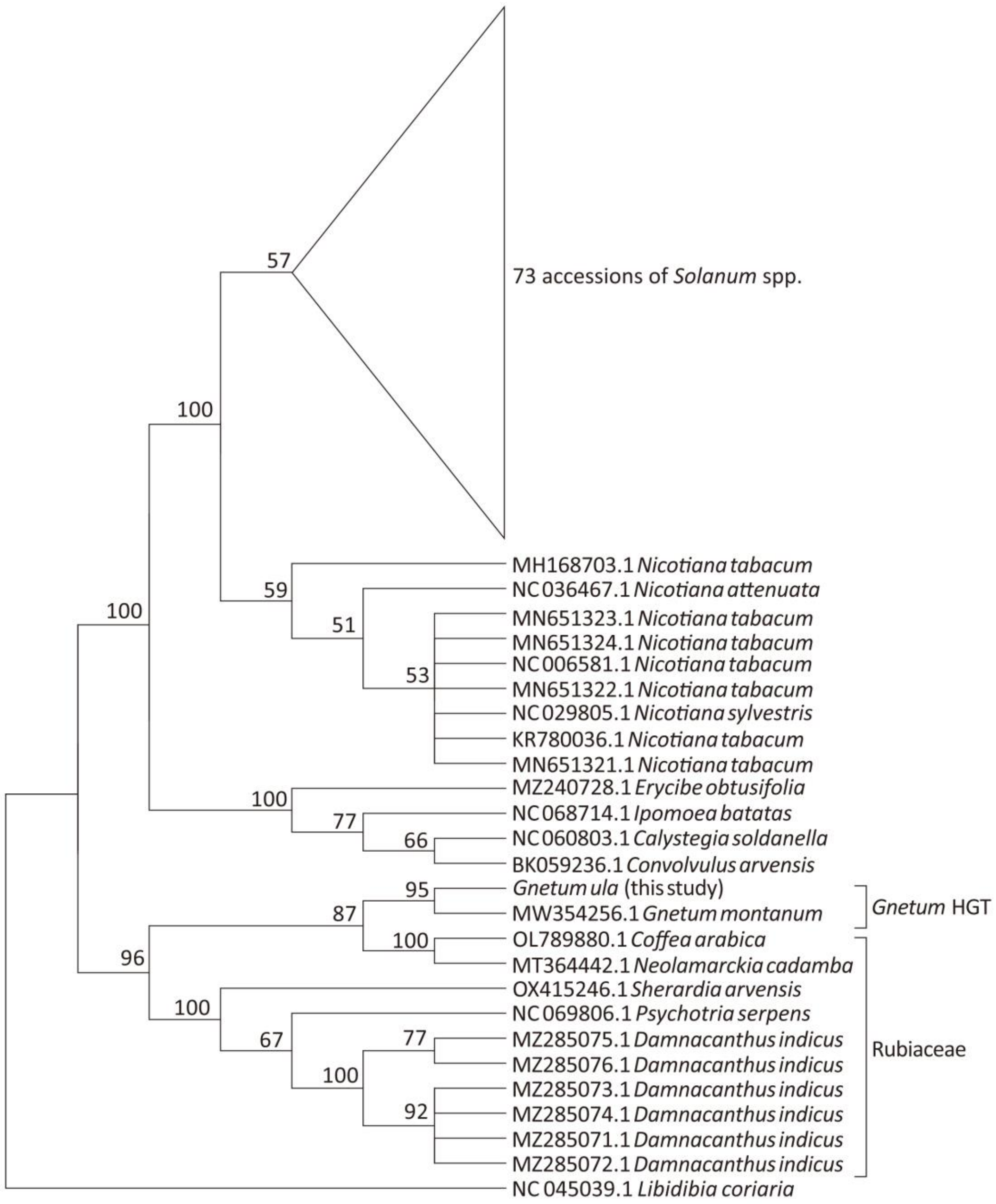

Fig. S23. A ML tree inferred from the flanking region of the 3’ end of the *Coptosapelta*-derived *ndhB* exon1 & intron locus and its mitochondrial homologies. The tree is condensed under a 50% majority rule.

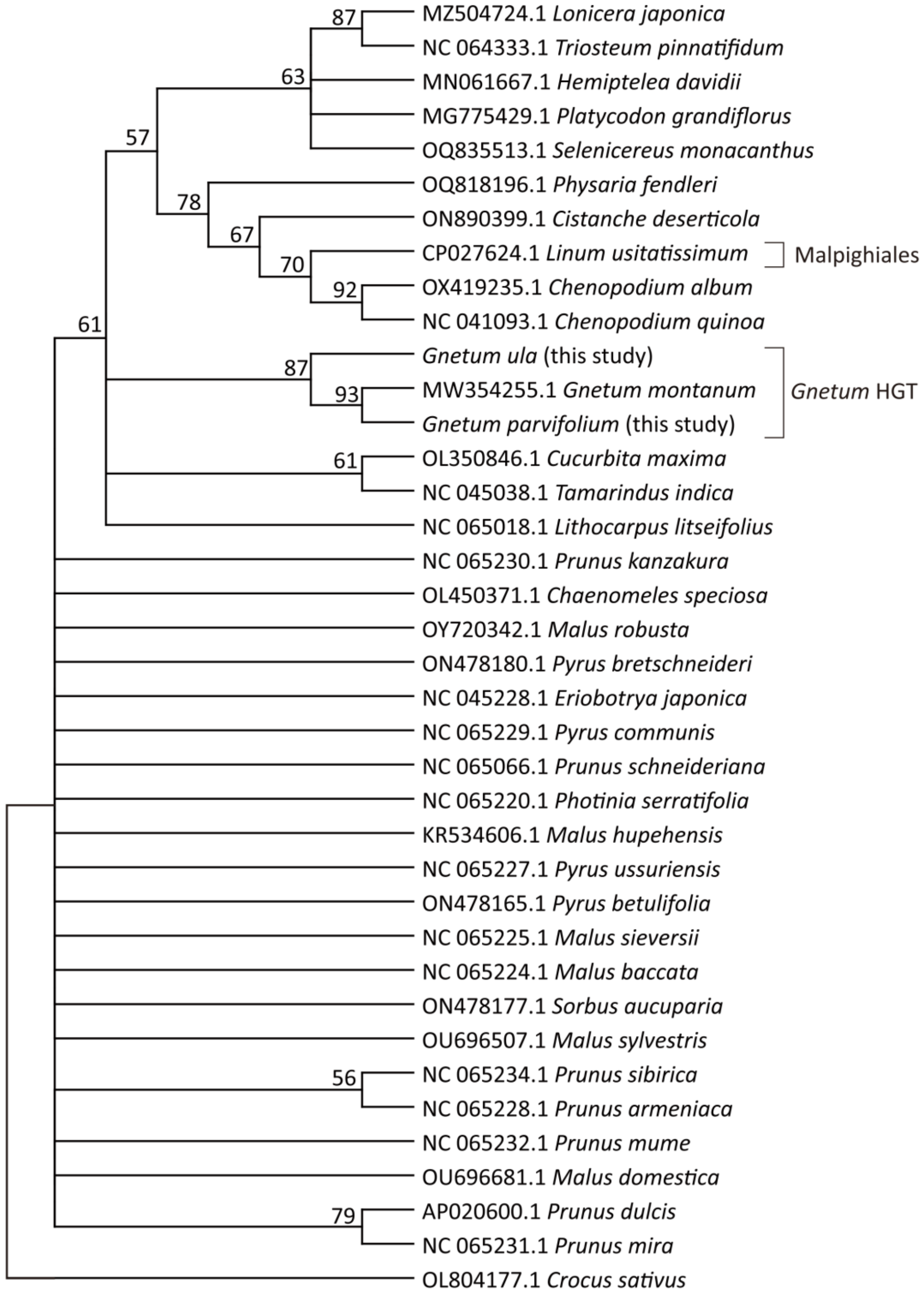

Fig. S24. A ML tree inferred from the flanking region of the 3’end of the Malpighiales-derived *psaB* locus and Its mitochondrial homologies. The tree is condensed under a 50% majority rule.
